# Machine learning to identify environmental drivers of phytoplankton blooms in the Southern Baltic Sea

**DOI:** 10.1038/s41598-025-85605-y

**Published:** 2025-01-24

**Authors:** Maximilian Berthold, Pascal Nieters, Rahel Vortmeyer-Kley

**Affiliations:** 1 Department of Biology, Faculty of Science, Mount Allison University, Sackville, Canada; 2 Institute of Cognitive Science, Osnabrück University, Osnabrück, Germany; 3 Institute for Chemistry and Biology of the Marine Environment, Carl von Ossietzky University Oldenburg, Oldenburg, Germany

**Keywords:** Scientific machine learning, Sparse identification of nonlinear dynamics, General additive mixed model, Baltic Sea, Phytoplankton blooms, Ecological modelling, Computational science, Scientific data

## Abstract

Phytoplankton blooms exhibit varying patterns in timing and number of peaks within ecosystems. These differences in blooming patterns are partly explained by phytoplankton:nutrient interactions and external factors such as temperature, salinity and light availability. Understanding these interactions and drivers is essential for effective bloom management and modelling as driving factors potentially differ or are shared across ecosystems on regional scales. Here, we used a 22-year data set (19 years training and 3 years validation data) containing chlorophyll, nutrients (dissolved and total), and external drivers (temperature, salinity, light) of the southern Baltic Sea coast, a European brackish shelf sea, which constituted six different phytoplankton blooming patterns. We employed generalized additive mixed models to characterize similar blooming patterns and trained an artificial neural network within the Universal Differential Equation framework to learn a differential equation representation of these pattern. Applying Sparse Identification of Nonlinear Dynamics uncovered algebraic relationships in phytoplankton:nutrient:external driver interactions. Nutrients availability was driving factor for blooms in enclosed coastal waters; nutrients and temperature in more open regions. We found evidence of hydrodynamical export of phytoplankton, natural mortality or external grazing not explicitly measured in the data. This data-driven workflow allows new insight into driver-differences in region specific blooming dynamics.

## Introduction

A fundamental understanding of ecosystem interactions is the foundation of a successful model^1^ e.g. of a phytoplankton bloom. Phytoplankton blooms vary in initiation timing, intensity, and annual recurrence. A meta-analysis of aquatic ecosystems worldwide revealed up to three different blooming patterns, characterized by the number of peaks per year^2^. These unique patterns are likely driven by a combination of abiotic factors (temperature, nutrients) and biotic factors (species composition, grazing)^3^.

However, predictions and forecasts of blooms can be obfuscated by global change and the rapid adaptation of phytoplankton communities to external drivers. Managed ecosystems, in particular, often experience rapid changes in external drivers to restore less disturbed or pristine conditions^4,5^. Tweddle et al.^6^ pointed out the importance of considering phytoplankton in marine management, as it is critical to the functioning of marine ecosystems, but at the same time has a large spatial and temporal patchiness that challenges management action plans. Monitoring data, used to create management action plans, often have low resolution (e.g. monthly sampling or coarse sampling in space) or lack crucial parameters (e.g. zooplankton grazing). This incomplete data makes it essential to learn as much as possible from available monitoring data for effective understanding and action. Describing drivers and interactions for different phytoplankton blooming patterns is challenging when key parameters are missing. Tools like general additive mixed models (GAMMs) efficiently describe patterns, but they do not necessarily enhance predictability or causality analysis, underscoring the need for new approaches.

Ecological sciences are thus increasingly turning to advanced data modelling tools. Pichler et al.^7^ listed various Machine Learning algorithms applicable to ecological questions, solving tasks in classification, regression, and object detection in the fields of decision making, species distribution, remote sensing, and other questions of ecosystem and biodiversity descriptions.

In the context of phytoplankton blooms, recent research has applied random forest approaches to identify driving factors of algal blooms and predict phytoplankton community structure^8,9^. Other studies have compared different machine learning methods to predict the development of harmful algal blooms and its consequences^10–12^.

Over the past decades, deep and artificial neural network (ANN) models have also been used to combine data-driven machine learning with knowledge of the underlying dynamic relationships of system variables^13–20^. This approach can enhance system dynamics understanding. However, a fundamental problem is that machine learning methods often function as “black boxes” and for example Wood^21^ recently pointed out the need for more transparent and explainable machine learning. It is especially difficult to interpret systematic relationships among variables directly. The SInDy (Sparse Identification of Nonlinear Dynamical Systems) algorithm by Brunton et al.^22^ addresses this issue. SInDy can translate the output of machine learning systems by identifying algebraic, interpretable combinations of basis functions through sparse linear regression, helping to understand underlying dynamics,^e.g.^^23,24^.

In this context, this work focuses on data-driven insights into the drivers of different phytoplankton blooming patterns with the overarching methodological question: *Is it possible to derive environmental drivers of phytoplankton blooms directly from monitoring data using a differential equation generating Machine Learning approach?* This question has two consequences for our understanding of the relationship between the data and the understanding of the observed system: On the one hand: *Is there knowledge hidden in the data that is so far overseen in other analysis?* On the other hand: *Is the found knowledge useful to inform management actions or modelling of the observed system?* Thus, we are explicitly combining the statistical description of data and the idea of mechanistic modelling of algal blooms in a single, overarching approach, taking a step towards explanatory predictions of ecosystems.

**Fig. 1:**
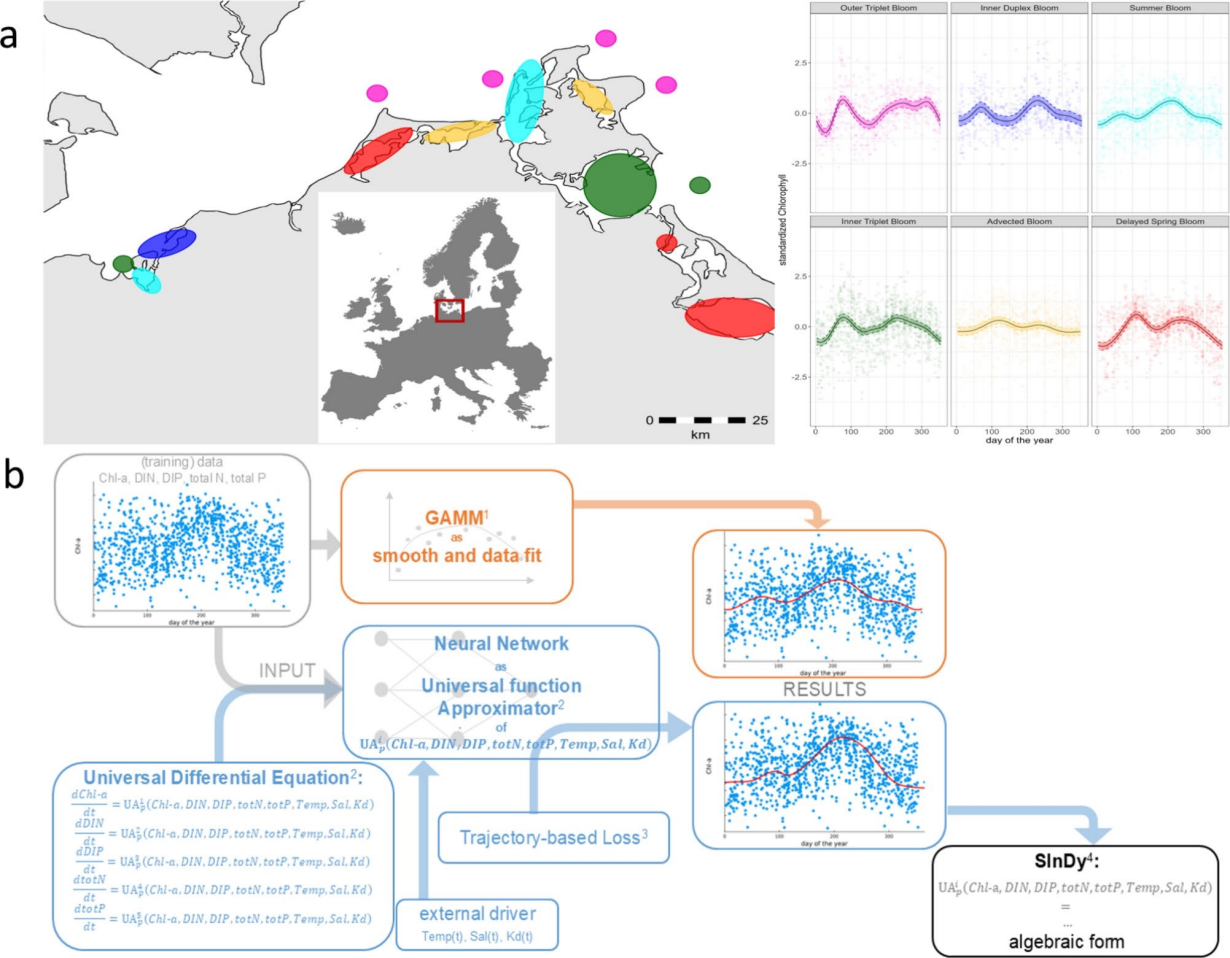
(**a**) Distribution of blooming patterns along the southern Baltic Sea coast at the northern European shelf. The color code of the blooming patterns (magenta: Outer Triplet Bloom, blue: Inner Duplex Bloom, cyan: Summer Bloom, green: Inner Triplet Bloom, yellow: Advected Bloom, red: Delayed Spring Bloom) correspond to the regions of occurrence. (Figure created using Microsoft PowerPoint 2013 and R-package ggplot). (**b**) Analysis concept for the data: log-transformed, z-scored data of chlorophyll-a (Chl-a), dissolved inorganic nitrogen (DIN), dissolved inorganic phosphorus (DIP), total nitrogen (totN) and total phosphorus (totP) in a Day-of-the-year time frame are modeled with a GAMM (1:^32^) (orange analysis path). Alternatively, they are used as training data for the Neural Network as an Universal function Approximator together with an uninformed Universal Differential Equation (2:^18^, 3:^31^) and water temperature Temp(t), salinity Sal(t) and light attenuation Kd(t) in a functional form as external drivers to approximate the underlying dynamics (blue analysis path). The SInDy approach (4:^22^) can be used to translate the output of the Neural Network analysis into an algebraic form.

As study example, we used a 22-year data set of the southern Baltic Sea coast, encompassing 30 monitoring stations and six different phytoplankton blooming patterns as identified in an earlier study by Berthold et al.^25^ (see Fig. 1a).

The Baltic Sea is a large brackish inland sea and is subject to frequent large scale phytoplankton blooms. The Baltic Sea currently shows a higher sea surface temperature anomaly than other water bodies in the world and is thus considered to provide useful predictions for a future ocean^26^. All riparian states of the Baltic Sea agreed to lower nutrient inputs, especially from nitrogen (N) and phosphorus (P), to curb the negative effects of phytoplankton blooms^27^. Coastal waters are heavily impacted by increased nutrient levels and subsequent eutrophication, as coastal waters can act as nutrient filters^28^.

Phytoplankton blooms remain frequent in the coastal waters of the southern Baltic Sea, exhibiting distinct blooming patterns even over relatively small geographical scales (200 km)^29^. Understanding their interactions within the ecosystem is critical for addressing mismatches between nutrient load reductions and insufficient decreases in phytoplankton biomass, potentially driven by hysteresis^5^. Improved knowledge of bloom drivers can inform management plans, such as the Baltic Sea Action Plan^30^, to better support coastal ecosystem health, with broad implications.

Our main contribution is the development and application of a new, multi-step method to find dynamical relationships of phytoplankton biomass, nutrients and external drivers from measurement data. The paper is organized as follows:

1. We used generalized additive mixed models (GAMM) to predict phytoplankton biomass development for the six different phytoplankton blooming patterns along the southern Baltic Sea coast described in Berthold et al.^25^ (see Fig. 1b orange path and Section “From monitoring data to Differential equations”).
2. We applied an extension by Vortmeyer-Kley et al.^31^ of the Universal Differential Equation (UDE) approach for Scientific Machine Learning^18^ to learn the coupled dynamic of chlorophyll-a (Chl-a), total nitrogen (totN), total phosphorus (totP), dissolved inorganic nitrogen (DIN), dissolved inorganic phosphorus (DIP) and the external drivers water temperature (Temp), salinity (Sal) and light availability (given as light attenuation coefficient Kd) for the different phytoplankton blooming patterns (see Fig. 1b blue path and Section “From monitoring data to Differential equations”). We refer to this approach as $\text {ANN}_\text {UDE}$ throughout the paper.
  This $\text {ANN}_\text {UDE}$ approach was able to match the performance of the GAMM (Section “Comparing GAMM and ANN_UDE_ results”). Additionally, we validated how well the models fitted the validation data (Section “Validation of phytoplankton blooming patterns”).
3. We applied the Sparse Identification of Nonlinear Dynamics (SInDy) algorithm by Brunton et al.^22^ to extract the interpretable dynamics from the UDE for each blooming pattern (see Fig. 1b and Section “SInDy’s description of driving factors”). When translating the $\text {ANN}_\text {UDE}$ output into a sparse second-order polynomial, SInDy preserved predictability while revealing key nonlinear terms driving phytoplankton blooms.
  Our analysis identified distinct combined nonlinear interactions influencing blooming dynamics across different patterns and seasons (Section “SInDy’s description of driving factors” and “Conclusions and Perspectives”, where also methodological difficulties and open questions of the approach are addressed).

The data-driven workflow we present predicts blooming pattern development while uncovering insights into dynamical relationships between nutrients, phytoplankton biomass, and external drivers. These relationships, acting as growth or loss terms, matched ecological knowledge on phytoplankton blooms in the region, and discovered new interactions that can inform models of coastal ecosystems. These insights thus also showcase the potential of our method to improve management actions.

Throughout the text, references to supplemental material are marked with an S and greatly help understand the complex interactions.

## From monitoring data to differential equations

The raw monitoring data was provided by the State Agency for Environment, Nature Conservation and Geology Mecklenburg-Vorpommern (LUNG-MV). This monitoring allows for example to follow up effects of local management actions as improved water treatment plants or to track the environmental status of the Baltic coastal waters, as a good environmental status is one goal of the Baltic Sea Action Plan^30^.

For our study, we used monthly data of chlorophyll-a (Chl-a), dissolved inorganic nitrogen (DIN), dissolved inorganic phosphorus (DIP), total nitrogen (totN), total phosphorus (totP), water temperature (Temp), salinity (Sal) and light attenuation coefficient (Kd, calculated from Secchi depth according to Liu et al.^33^ Eq. 17: $K_d=1.32\cdot \frac{1}{Secchi~depth}$) from 30 measurement stations along the German Baltic Sea coast, covering outer coastal waters, estuarine and marine lagoons, and shallow bays for the years 2000-2021. The data were sampled in the first two meters of the water column. Even though they were sampled monthly, they were not necessarily sampled on the same day of the month each year, resulting in a scattered data set on the day-of-the-year scale. The reason for the selection of the chosen variables was to represent the key nutrient:plankton dynamics under the constrains of external drivers. Thereby Chl-a acted as proxy for biomass, DIN and DIP as proxy for the main nutrients to be consumed and totN and totP served as a proxy for the overall nutrient status within the water column. Water temperature, salinity and light attenuation (as proxy for light availability) were selected as main external drivers.

All data were log-transformed, then z-scored, and split into a training data set (2000-2018) and a validation data set (2019-2021) (all data is available in the preprocessed format here

https://github.com/pnieters/PredictingPhytoplanktonPatterns). For the $\text {ANN}_\text {UDE}$ based analysis we dropped all time points where information on one variable was missing.

The GAMM was calculated for each parameter per day-of-the-year (orange path Fig. 1b). To close the annual cycle, we used cyclic cubic regression splines. We included sample year and sampling station as random factors and optimized the fit using restricted maximum likelihood^32^. The output of the GAMM was used to predict six different blooming patterns with coastal waters being grouped following wavelet transformation and changepoint analyses of an earlier study^25^. These patterns differed in number and amplitude of peaks and were sorted into: Outer Triplet Bloom (three blooms in outer coastal water), Inner Duplex Bloom (two blooms in inner coastal water), Summer Bloom (one bloom), Inner Triplet Bloom (three blooms in inner coastal water), Advected Bloom (moving blooms), Delayed Spring Bloom (shifted spring bloom in inner coastal water) (Fig. 1a). Importantly, blooming patterns were not constrained to one water body only, but the same blooming pattern occurred at different location along the coast. Furthermore, blooming patterns were not only sorted by number of peaks, but also level of Chl-a at peak time, as phytoplankton bloom peaks may be driven by different factors depending on trophic level.

Additionally, we trained an artificial neural network (ANN) to describe the temporal dynamics of the variables Chl-a, DIN, DIP, totN, and totP (blue path Fig. 1b).

We assume that the dynamics of these variables can be described by a set of differential equations depending on Chl-a, DIN, DIP, totN, and totP and some external driver, but the functional form of these equations is unknown (Box ”Universal Differential Equation” in blue path Fig. 1b). To uncover these differential equations an ANN was incorporated into the Universal Differential Equation (UDE) framework^18^ and trained to estimate the time derivatives of the variables based on their current values and the external drivers (Box ”Neural Network as Universal function approximator” in blue path Fig. 1b).

UDEs are trained by numerically simulating an ordinary differential equation where the time derivatives are given by the ANN’s output. The output of the simulated dynamics is then compared to training data using a prescribed loss function (Box “Trajectory-based Loss” in blue path Fig. 1b). The UDE approach uses automatic differentiation algorithms to compute gradients of the error signal - the difference between simulated and in training data observed dynamic - through both the numerical simulation and the ANN. These gradients are then used to update the ANN parameters, minimizing the error and ensuring that the simulated dynamic align closely with the measured training data.

In our case inputs to the ANN were the five variables Chl-a, DIN, DIP, totN, and totP measured in the Baltic Sea (grey Box ”training data” in Fig. 1b) along with three external drivers: Temp, Sal, and Kd. The functional form of the external drivers is a polynomial fit of the dynamics of the transformed Temp, Sal and Kd data for each blooming pattern from 2000 to 2018 together, creating a mean dynamic of these drivers. This mean-dynamic view is crucial because all analyses were conducted on a day-of-the-year scale for all training years combined The polynomial fit for water temperature is of order five, and for salinity and Kd, it is of order four (see supplemental Figs. S1–S3).

An ANN with 8 input neurons (5 neurons for the differential equation variables and 3 neurons for external drivers), 4 hidden layers with 16 neurons each and 5 output neurons (for the 5 differential equation variables) was trained for 500 iterations. Less complex neural networks failed to attain reasonable fits during pre-experimentation. The reason why neural networks can be fitted to data so effectively with a surprisingly large number of parameters is an ongoing debate in the deep learning community^34,35^.

The activation function was the Gaussian-Error-Linear-Unit (GELU) function^36^, weights were initialized using the Glorot intialization^37^ and the LDA loss function^31^ with hyperparameter 0.2 (0.8) for the length difference (angle difference) was used.

**Fig. 2:**
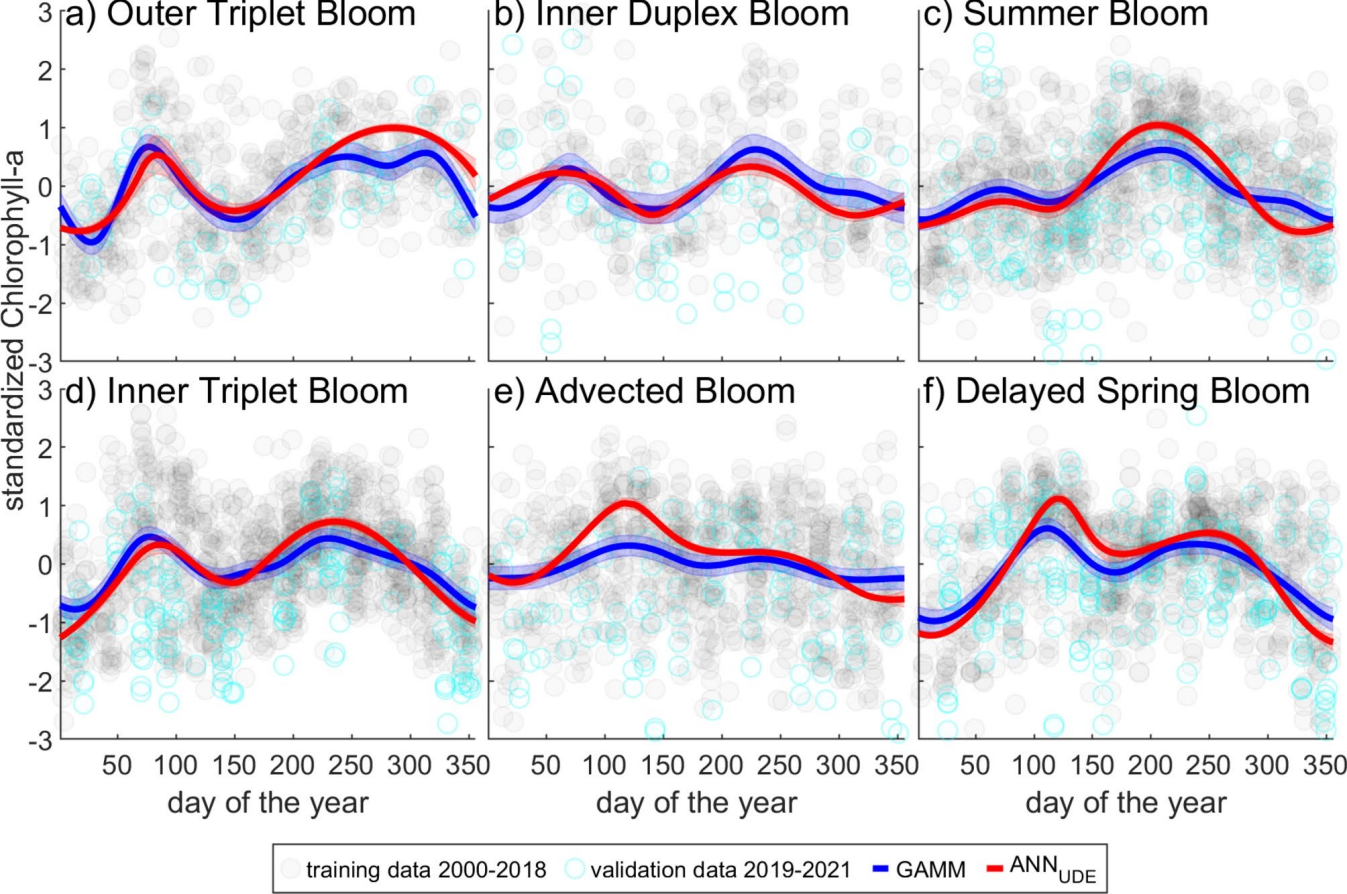
Standardized chlorophyll-a dynamics of the different blooming patterns: (**a**) Outer Triplet Bloom, (**b**) Inner Duplex Bloom, (**c**) Summer Bloom, (**d**) Inner Triplet Bloom, (**e**) Advected Bloom, (**f**) Delayed Spring Bloom. Light gray dots: log-transformed, z-scored data of Chlorophyll-a measured at the 30 stations between 2000-2018 used as training data. Cyan dots: log-transformed, z-scored data of Chlorophyll-a measured at the 30 stations between 2019-2021 used as validation data. blue: GAMM approximation with confidence interval based on two times standard error, red: mean-model of the $\text {ANN}_\text {UDE}$ approach with confidence interval based on two times standard error.

Due to the random weight initialization of the ANN, models varied slightly as a result of training^31^. Therefore, we trained 20 ANN for each blooming pattern and calculated a mean of each set of results (ensemble approach). The mean model, together with the solution for the GAMM, is presented in Fig. 2 (for the full sets of results see Figs. S4–S9 in Supplementary Information).

We used the mean squared error (MSE) to evaluate how well the two modelling approaches described the data. $x_i$ are the data points of the data set with *N* data points in the time interval and $m_i ^K$ is the model prediction of the model type *K*, MSE is given as

1 $$\begin{aligned} \textrm{MSE}_{data-model} = \frac{1}{N}\sum _{i=1}^{N}{(m_i^K - x_i)^2} \end{aligned}$$

Equation (1) can be rewritten as follows to compare the results of GAMM and $\text {ANN}_\text {UDE}$:

2 $$\begin{aligned} \textrm{MSE}_{GAMM-ANN_{UDE}} = \frac{1}{N}\sum _{i=1}^{N}{(m_i^{GAMM} - m_i^{ANN_{UDE}})^2} \end{aligned}$$

All data and code used in this study can be found in a public repository here

https://github.com/pnieters/PredictingPhytoplanktonPatterns.

## Comparing GAMM and $\text {ANN}_\text {UDE}$ results

Interestingly, both the GAMM and the mean model of the $\text {ANN}_\text {UDE}$ showed similar blooming patterns, with some cases of slightly shifted blooming peak times (Fig. 2). Comparing the $\text {ANN}_\text {UDE}$ mean model and the GAMM model (Eq. 2) resulted in MSE values ranging from 0.04 to 0.11, indicating high similarity between the models.

The $\text {MSE}_{data-model}$ (Eq. 1) showed values ranging from 0.62 to 0.92 for different blooming patterns descriptions, suggesting similar model quality in both approaches (see Table S1 in Supplementary Information). However, the MSE values for the data-GAMM-comparison were consistently slightly lower than those for the data-$\text {ANN}_\text {UDE}$-mean-model-comparison. The reason could that the GAMM was able to capture high variability in the data using the flexibility of spline fitting, where the $\text {ANN}_\text {UDE}$ produced a close-form description in the form of a differential equation, which smoothed out high variability a bit.

## Validation of phytoplankton blooming patterns

We evaluated the phytoplankton blooming patterns we found using data for the years 2019–2021. The MSE values were slightly higher (1.08–1.33 for data-$\text {ANN}_\text {UDE}$ and 1.14–1.37 data-GAMM-comparison) for four of the six blooming patterns (see Supplementary Information Table S2 for details). The slightly lower MSE values for the data-$\text{ANN}_\text {UDE}$ models on the validation data set supports our interpretation that the spline approach of the GAMM model might slightly overfit the data.

Exceptions in the MSE were the Outer Triplet Bloom (0.66 for data-$\text {ANN}_\text {UDE}$ and 0.51 data-GAMM-comparison), and the Advected Bloom (2.46 for data-$\text {ANN}_\text {UDE}$ and 1.94 data-GAMM-comparison), indicating that external drivers temperature and salinity affected the validation data sets for these water bodies.

The close approximation of the validation data for the Outer Triplet Bloom could be due to ongoing exchange with the open sea, which might buffer temperature effects for these blooms in exceptionally warm years. On the other hand, temperature is a growth-supporting term for the Outer Triplet Bloom during their summer blooming period (see Section “SInDy’s description of driving factors”). Consequently, this term would still lead to a Chl-a increase in exceptionally warm years if phytoplankton communities can acclimatize to higher temperatures in these coastal waters.

In contrast, the Advected Bloom appeared to be dependent on nutrient dynamics, with nutrients entering and leaving the semi-enclosed coastal regions through flushing events as discussed above (see Section “SInDy’s description of driving factors”). These events can change during exceptional warm and dry years.

## SInDy’s description of driving factors

The $\text {ANN}_\text {UDE}$ approach successfully recovered the blooming patterns described by the GAMM. Our primary objective, however, was to gain insight into how these different blooms were driven by both linear and nonlinear interactions within the system’s variables and external drivers. To achieve this, we applied the SInDy algorithm^22^, which approximates the $\text {ANN}_\text {UDE}$ solution using a simpler, more interpretable functional form compared to the black-box ANN (last box of blue path Fig. 1b).

SInDy uses sparse linear regression to identify governing equations by approximating the time derivatives of a dynamical system using a set of nonlinear basis functions. In our study, we selected polynomial combinations of variables and external drivers up to the second order - e.g., Chl-$\hbox {a}^2$, $\text{Chl-a}\cdot\text{DIN}$, $\text{Chl-a}\cdot\text{Kd}$ - resulting in 45 total functions in our basis function library (for comments on this choice see Section “Conclusions and perspectives”).

The resulting model took the form:

3 $$\begin{aligned} {\frac{ \text{ d} \text{Chl-a }}{\text{ dt }}}= \partial _t \text{ Chl-a } = c_1 + c_2\cdot \text{ Chl-a } + c_3\cdot \text{ Chl-a}^2 + c_4\cdot \text{ DIN } + c_5 \cdot \text{ Chl-a }\cdot \text{ DIN } +...+c_{45}\cdot \text{ Kd}^2 \end{aligned}$$

where $c_i$ were the linear coefficients associated with the basis functions.

Because we chose to fit a $\text {ANN}_\text {UDE}$ model first, we were able to use the $\text {ANN}_\text {UDE}$ to approximate the time derivative and then used this approximation to train the SInDy model, avoiding direct estimation of derivatives from very noisy data. Specifically, out of the ensemble of 20 $\text {ANN}_\text {UDE}$ models, we chose the $\text {ANN}_\text {UDE}$ that matched the ensemble mean the best (smallest MSE, see supplemental Figs. S10–S15 for chosen models) per blooming pattern. SInDy then applied a linear penalty (LASSO, see Brunton et al.^22^ for details) to fit a sparse model, balancing the number of nonzero coefficients with the quality of the model’s fit. This resulted in a final solution where most of the terms in the governing equation were zero, leaving only the most significant coefficients and basis functions as indicators of the linear and nonlinear dependencies driving specific blooming patterns. We tuned the sparsity coefficients to achieve an optimal trade-off between sparsity and model accuracy (see code for details).

**Fig. 3:**
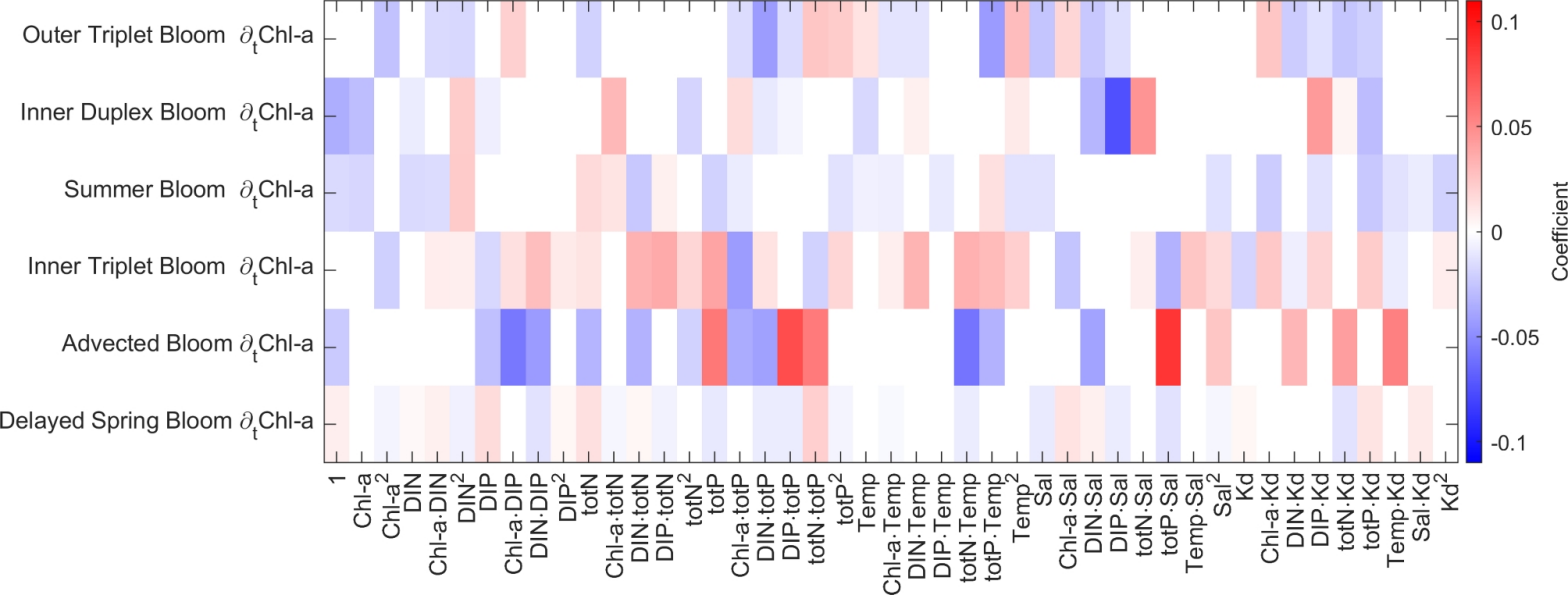
Selection of basis functions of the differential equations of Chl-a sorted by bloom type and ordered lowest (Outer Triplet Bloom) to highest mean Chlorophyll-a concentrations (Delayed Spring Bloom). The coefficient denotes the positive or negative impact of a basis function on $\partial _t$Chl-a for a given time point (cf. Eq. 3). However, the overall sign of the terms depend on the actual value of the state variable (above or below-average values). The full set of differential equations for all phytoplankton blooming patterns can be found in Supplementary Information Fig. S16. The dynamic of the different terms $\text{coefficient}\cdot\text{basis function}$ over the course of the year in the differential equation for Chl-a for the different phytoplankton blooming patterns are shown in Fig. 4.

Already our small set of basis functions provided an accurate description of the different blooming patterns (residual sum of squares between 2.82$\cdot 10^{-5}$ and 1.78$\cdot 10^{-3}$, details see SindyOUTallEQ.txt at https://github.com/pnieters/PredictingPhytoplanktonPatterns) and their different driving factors (see Fig. 3 for each differential equation for Chl-a. The full set of differential equations can be found in the Supplementary Information Fig. S16).

It is important to note again that the analysis is based on log-transformed and z-scored data, when interpreting and discussing the differential equations found, allowing only to describe changes above or below the average. Thus, no direct conclusions about quantitative biomass can be made.

Generally, terms in Eq. 3, expressed as $\text{coefficient}\cdot\text{basis function}$, with an overall positive effect are considered growth terms, leading to an increase in Chl-a, while an overall negative effect signifies loss terms (potential limitation or mortality), leading to a decrease in Chl-a.

An overall positive effect can result from negative coefficients multiplying a negative z-score (below-average values) of a variable, or from positive coefficients multiplying a positive z-score (above-average values) of a variable. In higher order polynomials, the overall effect depends on the sign of the polynomial product and the sign of the coefficient. For example, the product of two variables, each with negative z-score, combined with a positive coefficient results in a positive overall effect, indicating Chl-a growth. A table detailing all possible combinations of signs of state variables, basis functions and coefficients, and whether they indicate growth or loss terms, is given in Table S3 in Supplementary Information.

**Fig. 4:**
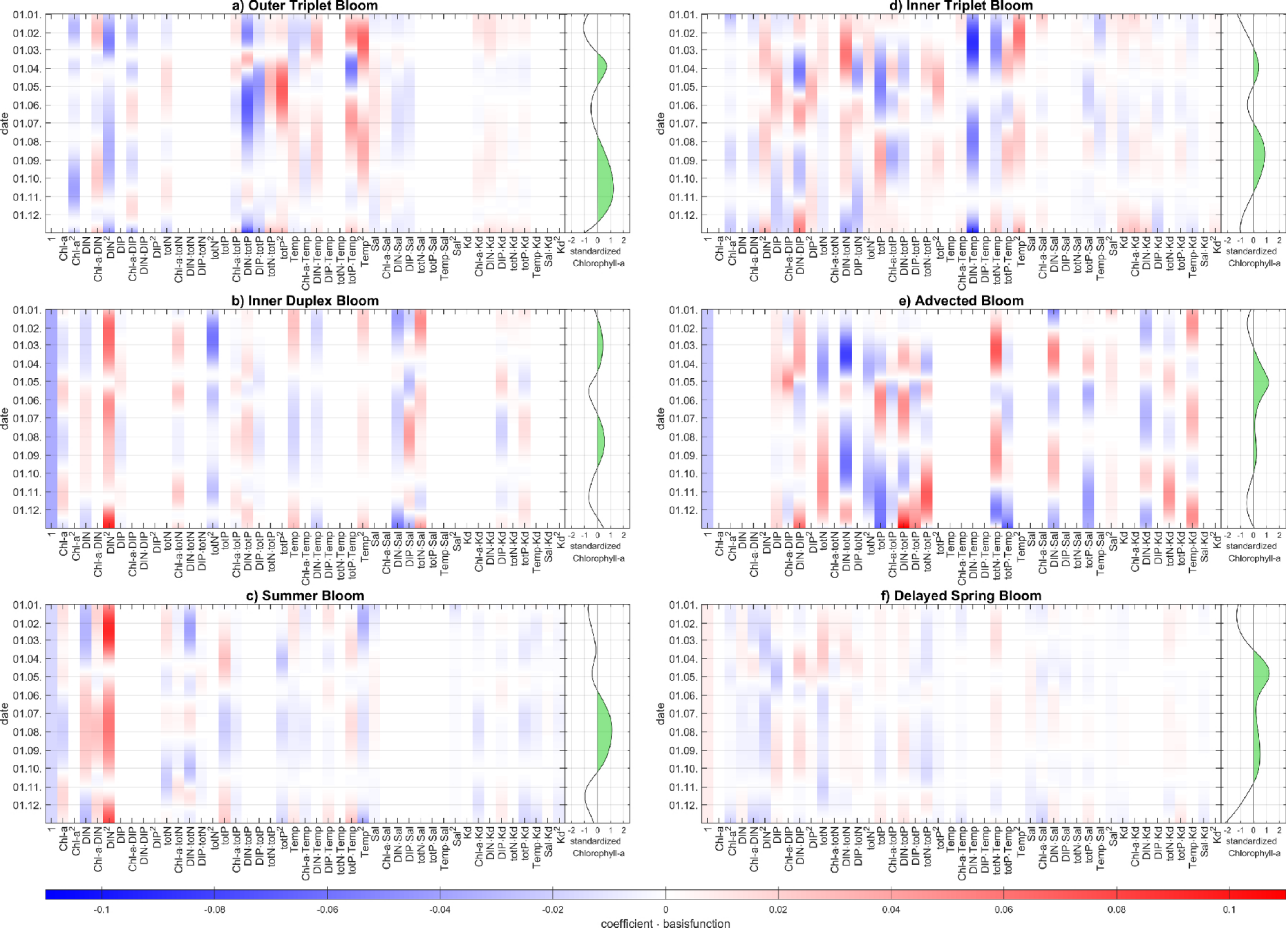
Temporal impact of the $\text{coefficient}\cdot\text{basis function}$ values (x-axis) in the differential equation for Chl-a (heatmap (left)) over the course of the year (y-axis) in comparison to the chlorophyll-a dynamics (timeseries plot (right), above average Chl-a periods marked green) for: (**a**) Outer Triplet Bloom, (**b**) Inner Duplex Bloom, (**c**) Summer Bloom, (**d**) Inner Triplet Bloom, (**e**) Advected Bloom, (**f**) Delayed Spring Bloom.

Since we were interested in the driving factors for each blooming pattern, in the following discussion we will focus only on Chl-a during periods when the Chl-a z-score was positive (indicating above-average concentrations) and increasing, representing growing phytoplankton blooms.

To simplify the results presentation, we will focus on main drivers, like DIN, DIP, totN, totP, temperature, salinity, and Kd, as well as on the most positive and most negative terms, but not on all potential interaction terms of them found in the differential equations for Chl-a and exemplarily discuss important terms. Table 1 summarizes the terms with strongest positive and negative contributions per bloom respectively. For positive and negative contributions, we also give the respective proportion each term covered within the group of positive or negative contributions during the growth period of each bloom.

To make the description of the different terms more readable, we use ”negative basis function” as short form for ”basis function multiplied with a negative coefficient” and ”positive basis function” as short form for ”basis function multiplied with a positive coefficient”.

**Table 1:** Contribution of most positive and most negative basis functions during growing phytoplankton blooms.

Blooming pattern	Most positive basis function	Most negative basis function
Outer Triplet Bloom	Bloom 1: $\hbox {Temp}^2$ (0.30)	Bloom 1: $\text{totP}\cdot\text{Temp}$ (0.39)
	Bloom 2: $\hbox {Temp}^2$ (0.28)	Bloom 2: $\hbox {DIN}^2$ (0.34)
Inner Duplex Bloom	Bloom 1: $\hbox {DIN}^2$ (0.37)	Bloom 1: $\hbox {totN}^2$ (0.32)
	Bloom 2: $\text{DIP}\cdot\text{Sal}$ (0.26), $\hbox {DIN}^2$ (0.25)	Bloom 2: constant (0.27)
Summer Bloom	Bloom 1: $\hbox {DIN}^2$ (0.43)	Bloom 1: constant (0.16), totP (0.15)
Inner Triplet Bloom	Bloom 1: $\text{DIN}\cdot\text{totN}$ (0.23)	Bloom 1: $\text{DIN}\cdot\text{Temp}$ (0.30)
	Bloom 2: $\hbox {Temp}^2$ (0.14), totP (0.14)	Bloom 2: $\text{DIN}\cdot\text{Temp}$ (0.40)
Advected Bloom	Bloom 1: $\text{totN}\cdot\text{Temp}$ (0.21)	Bloom 1: $\text{DIN}\cdot\text{totN}$ (0.29)
	Bloom 2: $\text{totN}\cdot\text{Temp}$ (0.34)	Bloom 2: $\text{DIN}\cdot\text{totN}$ (0.37)
Delayed Spring Bloom	Bloom 1: $\text{DIN}\cdot\text{DIP}$ (0.20), totN (0.19)	Bloom 1: DIP (0.27)
	Bloom 2: $\text{DIN}\cdot\text{DIP}$ (0.16)	Bloom 2: $\hbox {DIN}^2$ (0.28)

The highest positive and negative contributions from all basis functions were identified by integrating all their contribution during periods when the Chl-a was positive and increasing. We then identified the highest contribution among positively contributing basis functions and negatively contributing basis functions respectively. The numbers in the brackets indicate proportion of the contribution within the group of positive or negative contributions respectively. If two basis functions contribute equally or nearly equally both are listed.

The **Outer Triplet Bloom** was characterized by 24 active basis functions (Figs. 3, 4a).

Only the basis function for total nitrogen (totN) was active (non-zero) and negative among all main linear nutrient factors. Thus, the linear totN basis function would act Chl-a suppressing if totN showed above-average concentrations (positive z-score), and vice versa. We found that below-average totN concentrations coincided with higher Chl-a concentrations during the first bloom event in March/April and the long blooming period from mid-September to mid-November. Likewise, the negative quadratic DIN term had a growth-suppressing effect during the onset of the first bloom and was also the most negative term during the second bloom (cf. Table 1 ). Contrary, the positive quadratic totP term had a growth enhancing effect during the same period, indicating a reliance of the bloom on P rather than N.

Interestingly, the Outer Triplet Bloom showed a loss term coupled to the square of Chl-a, indicating natural mortality or grazing depending on the amount of available Chl-a, thereby regulating the bloom. Such quadratic loss terms depending on the amount of plankton are sometimes used in NPZ models (nutrients-phytoplankton-zooplankton)^38,39^.

The abiotic factors temperature, and salinity also showed active basis functions. The linear temperature term included a positive coefficient within the differential equation, thus negative z-scores (below-average temperature) would lower the amount of Chl-a. Interestingly, the quadratic temperature term was Chl-a growth supporting, potentially indicating that rapid temperature changes still act bloom enhancing. We found that below-average temperatures lowered the amount of Chl-a during the first bloom from March to mid-April, while above-average temperatures supported Chl-a during the long blooming period from July to November. This dynamic was further accompanied by a consistently most positive quadratic temperature term promoting Chl-a growth during both blooms. This accounted for on third of the total positive contribution during both bloom’s growth phase (cf. Table 1). Thus phytoplankton growth is potentially temperature-limited in these coastal waters. This finding aligns with long-term observations where phytoplankton blooms in these coastal waters start earlier, potentially triggered by elevated temperatures^40^.

The linear salinity term included a negative coefficient, indicating that a positive log-transformed, z-score (above-average salinity) lowered Chl-a concentrations. We found that below-average salinity concentrations supported Chl-a from the peak of the first bloom in March until the onset of the second bloom in September. Conversely, above-average salinity suppressed Chl-a during the blooming period from October to November. A possible explanation is that local phytoplankton populations thrive in lower salinity water than usually found in these coastal waters^41^ coming from adjacent, more eutrophic coastal waters. Another potential explanation is that this incoming water carries more Chl-a into the outer coastal waters, mimicking a phytoplankton increase by dilution from higher Chl-a areas.

In summary the largest impact on the formation of the blooming pattern of the Outer Triplet Bloom were given by temperature and nutrients as they are linked to the most negative and most positive terms during the blooming period (cf. Table 1).

The **Inner Duplex Bloom** was characterized by the smallest number of active basis functions (19 basis functions) (Figs. 3, 4b).

Both dissolved nutrient fractions, linear DIN, quadratic DIN and DIP, were active basis functions. The negative linear DIN basis function suppressed Chl-a in case of above-average DIN concentrations, like during the first bloom. However, the positive quadratic DIN term was stronger and potentially overwrite the effects of the negative linear DIN term during this phase resulting in an overall growth enhancing effect of DIN. Additionally, the quadratic DIN term contributed most to both bloom growth phases and this effect was larger during the first bloom (cf. Table 1). Conversely, below-average DIN could result from its utilization by the bloom, supporting high Chl-a, or from the initiation of an N-fixing cyanobacterial bloom, which is frequently recorded in these coastal waters during summer^29^.

The presence of a linear and a quadratic term in DIN might suggest a Holling type II^42^-like nutrient dependence for phytoplankton growth. The Taylor expansion of Holling type II would include linear as well as quadratic terms and such Holling type II-like terms are one possible way to model nutrient dependence in NPZ-models^38^.

Likewise, the coefficient of the linear DIP basis function was also negative, pointing to similar mechanisms as with DIN. DIP had no effect on Chl-a during the first bloom, as winter/spring concentrations on average met phytoplankton demand. In contrast, above-average DIP during the second bloom also reduced the amount of Chl-a. This result is surprising, as both linear DIN and DIP did not directly support a Chl-a bloom.

However, compared to winter, highest precipitation is usually recorded during summer months in this coastal $\hbox {areas}^{\mathrm {e.g.}}$^43^. The resulting high runoff during summer could also increase DIN and DIP while diluting down phytoplankton blooms. This finding correlated with the interaction term of $\text{DIP}\cdot\text{Kd}$, combined with a positive coefficient. The interaction of $\text{DIP}\cdot\text{Kd}$ acted negatively on Chl-a, explained by above-average (positive z-score) DIP concentration, below-average Kd values and a positive coefficient. The below-average light attenuation indicated higher light availability and less turbid waters, potentially due to high DIP riverine inflow during summer. This finding was further corroborated by the interaction term of $\text{DIP}\cdot\text{Sal}$ with a negative coefficient. Thus, below average (negative z-score) salinity, above-average (positive) DIP and a negative coefficient supported Chl-a blooms during the second bloom. The most negative term during the first bloom was $\hbox {totN}^2$ (cf. Table 1).

In contrast to the Outer Triplet Bloom the Inner Duplex Bloom had a constant loss term, indicating natural mortality or grazing independent of the Chl-a amount. The constant loss was also the most negative term during the second bloom accounting for about one third of negative contributions during bloom growth (cf. Table 1). This may indicate a zooplankton population not limited by temperature grazing on phytoplankton. During the blooming period the linear Chl-a term acted as a linear loss factor. Usually, such loss factors are inserted on purpose in NPZ-models to model plankton based losses^38^.

Temperature was the only active abiotic factor showing a negative linear basis function. A below-average temperature supported the first bloom, and likewise an above-average temperature suppressed Chl-a during the second bloom. Temperature dependence in NPZ-models can be modeled in various ways, as reviewed by Grimaud et. al.^44^, where most of the approaches can be approximated locally by a linear and/or quadratic temperature term, as found in our SInDy approximation. The impact of the linear temperature term was accompanied by a consistently positive quadratic temperature term supporting Chl-a growth, enhancing the positive effect of the linear temperature term during the first bloom and partly balancing the negative effect of the linear term during the second bloom. The effect weakened at temperatures well above the average, while the overall effect of temperature for the second bloom was growth-suppressing.

These results indicate that the phytoplankton community in these coastal waters is more acclimatized to colder waters from the open Baltic Sea rather than warmer lagoon water from inner coastal waters. This is also supported by the fact, that $\text{DIP}\cdot\text{Sal}$ was the most positive term during the second bloom (cf. Table 1) potentially pointing to an impact of saltier more nutrient rich open Baltic Sea water. Usually, water from the open Baltic is not exhausted of DIP even during summer months^45^.

In summary the largest impact on the formation of the blooming pattern of the Inner Duplex Bloom were given by nutrients as the most negative and most positive terms during the blooming period are linked to them. Additionally, the second blooming period was also impacted by a constant loss (cf. Table 1).

The **Summer Bloom** was characterized by 25 active basis functions (Figs. 3, 4c).

DIN, but not DIP, occurred as an active linear term in the SInDy-derived differential equation. The negative linear DIN basis function acted as loss term for Chl-a, when DIN showed above average concentrations. Below-average DIN concentrations supported Chl-a growth during the sole bloom event in summer. This effect was enhanced by the always positive quadratic DIN term, which was also the most positive term for this blooming pattern accounting for about 40% of the positive terms’ impact (cf. Table 1). If below-average DIN concentrations caused such a bloom, then N-fixing cyanobacteria, which occur frequently in this area in summer^46^, could most likely be the cause. This interpretation is supported by the positive linear basis function of totN. Thus, above-average totN enhanced Chl-a growth, or just totN increased through N-fixation in phytoplankton biomass. Conversely, also a negative linear basis function in totP was found. Thus, above-average totP concentrations acted as one of the most negative loss term for Chl-a during the entire blooming period (cf. Table 1), accompanied by a negative quadratic totP term with a growth-suppressing effect. Increased totP concentrations could originate from sediment remineralization, or influx from the adjacent catchment area. Grazing would keep the totP fraction near constant, and not cause above-average totP concentrations. An influx of nutrient rich water from the catchment area and increased water exchange including a transport of Chl-a may explain why above-average totP act as a loss term for Chl-a in these coastal waters.

Like the Inner Duplex Bloom, the Summer Bloom showed a constant loss term indicating natural mortality, zooplankton grazing or hydrodynamical export of Chl-a. As was the case for the Inner Duplex Bloom, this term was one of the highest negative contributors here too (cf. Table 1). During the blooming season a negative linear term in Chl-a further limited Chl-a growth, depending on the total amount of Chl-a. These two terms were the other most negative terms during the blooming period. They indicated that the bloom is potentially light-limited, as more phytoplankton biomass shades itself. This finding is further supported by the negative $\text{Chl-a}\cdot\text{Kd}$ interaction term that also showed a growth-suppressing impact on Chl-a.

Temperature and salinity were both negative linear basis functions. Interestingly, above-average temperature acted as loss term for Chl-a during the summer bloom, as well as the always negative quadratic temperature term. Two possible explanations are that higher temperatures increase the activity of grazing zooplankton^47^, or that above-average temperature fall out of the growth optimum of locally adapted phytoplankton groups. However, the main component of phytoplankton in this area are (trichome) cyanobacteria, which are often better adapted to higher temperatures. Main grazers of such trichome cyanobacteria are rotifers in the Baltic Sea^48^, which may be more active at above-average temperatures^49^. This explanation could also be linked to the constant loss term and the linear loss in Chl-a during the blooming season described above pointing to possible external grazing.

In summary the largest impact on the formation of the blooming pattern of the Summer Bloom was given by nutrients and external loss regulation, as the most negative terms are linked to a constant and totP and the most positive terms to nutrients and DIN especially (cf. Table 1).

The **Inner Triplet Bloom** was the bloom characterized by the highest number of active basis functions (33 basis functions) (Fig. 3, Fig. 4d).

However, as with the Outer Triplet Bloom, the third bloom peak is not distinguishable in all runs and averaged into one big second bloom (See discussion on smoothing in Section “Conclusions and perspectives” and Fig. 2a,d, S4 and S7).

DIP, but not DIN was a negative linear basis function in these coastal waters. Below-average DIP acted positive on Chl-a growth during the first bloom event from early March to end of April. Conversely, above-average DIP concentrations acted as a loss term during the second and potential third bloom from August until November. During this period the quadratic DIP term still acted as growth-supporting and dampened the loss term effect of the linear DIP term especially for DIP well above the average. The overall effect of DIP was still growth-suppressing. The presence of a linear and a quadratic term in DIP might indicate a Holling type II-like nutrient dependence, similar to the one for DIN found for Inner Duplex Bloom type coastal waters.

DIP concentrations were around $0.6\mu \hbox{mol}$ $\cdot$ $\cdot\hbox{l}^{-1}$ during autumn and winter, with much lower concentrations of around $0.1\mu \hbox {mol}\cdot\hbox{l}^{-1}$ during spring^29^. Thus, above-average DIP concentrations likely indicate inflow from nearby estuaries, which can carry high loads of dissolved nutrients^50^, potential grazing, or P-remineralization during winter.

The total nutrient fractions of totN and totP were both associated with positive linear basis functions. Above-average totN concentrations supported, or correlated with Chl-a during the first bloom in spring and was also associated in combination with DIN as most positive term during this period. Conversely, below-average totN concentrations acted as loss term on Chl-a during the consecutive bloom events, indicating possible overall N limitations within the system. Indeed, totN concentrations are lowest during autumn ($32\mu \hbox {mol}\cdot\hbox{l}^{-1}$), compared to higher concentrations throughout winter and spring (up to $39\mu \hbox {mol}\cdot\hbox{l}^{-1}$)^29^. Below-average totP concentrations acted as loss terms on Chl-a during the spring bloom in these coastal waters, whereas above-average totP concentrations supported Chl-a during the later blooming periods, where it was also the most positive term (cf. Table 1). However, also a simple co-linearity with increasing phytoplankton biomass, which accumulated totP over the season is possible. P-depositional rates are highest during summer months in these coastal waters^51^, and accumulation was observed in mesocosm experiments without sufficient grazing control^25^. These results indicate that low P-availability may impact the first bloom, as observed with only $1\mu \hbox {mol}\cdot\hbox{l}^{-1}$ totP during spring, compared to $\sim$ $1.5\mu \hbox {mol}\cdot\hbox{l}^{-1}$ later in the year^29^. The effects of the always positive quadratic totN and totP terms were weak compared to the linear terms in totN and totP. The most positive term during the first bloom was $\text{DIN}\cdot\text{totN}$ (cf. Table 1).

Like the Outer Triplet Bloom, the Inner Triplet Bloom showed a quadratic negative term in Chl-a pointing to an increasing limitation of Chl-a with its increasing biomass.

The attenuation coefficient Kd was the only active abiotic factor associated with a negative linear linear basis function with different impacts on the two blooming periods: A below-average Kd (high light penetration) positively impacted Chl-a during the first bloom, however an above-average Kd acted as loss term on Chl-a (light limitation) for the remainder of the year. These results indicate that light availability, becomes a limiting factor for Chl-a particularly during spring, but also later in the year.

Contrary, the always positive quadratic temperature term acted growth-supporting, especially during the onset of the blooms. This interaction could be interpreted as a potential bloom starting but not bloom regulating effect. The most negative term during both blooming periods was the $\text{DIN}\cdot\text{Temp}$ term accounting for 30% of all negative contributions during during the first blooming period and for 40% during the second blooming period respectively (cf. Table 1).

In summary the formation of the blooming pattern of the Inner Triplet Bloom were governed by an interplay of nutrients, as the most positive and most negative terms are linked to nutrients and nutrient interaction terms (cf. Table 1).

For the **Advected Bloom** 20 active basis functions were identified (Figs. 3,  4e).

The advected bloom starts growing 2–4 weeks later than any other identified blooming pattern, and reaches its peak in May. An initial study linked this late bloom peak to the close location of such coastal waters to hypertrophic coastal waters where flushing of Chl-a-rich water may create an advected bloom^25^.

DIP was the only dissolved nutrient fraction associated with a negative linear basis function. Thus, a below-average DIP concentration coincided with increased Chl-a concentrations during the first bloom in April, indicating DIP uptake and subsequent growth. Conversely, above-average DIP concentrations acted as a weak loss term on Chl-a during the second bloom in August/September. The above-average concentration of DIP could indicate increased zooplankton grazing and thus release of DIP^52^, which might be linked to the active constant loss term interpreted as natural mortality, zooplankton grazing or the loss of Chl-a in the area of interest through hydrodynamical transport. Indeed, increased flushing caused by high precipitation during summer in these coastal areas^43^ could lead to increased DIP influx.

totN was associated with a negative active basis functions, while totP showed a positive one. totN acted as loss term on Chl-a during above-average totN concentration during the first bloom in spring. Together with the always negative quadratic totN term, this dynamic potentially indicates that outflow of nutrient rich waters from the catchment area flush out Chl-a^53^. This flushing could be associated with the $\text{DIN}\cdot\text{totN}$ term, which was the most negative term during both blooms and further increased in importance during the second bloom (cf. Table 1). Thus, the regulation of this blooming pattern might rather be governed by hydrodynamics than nutrient availability. Interestingly, below-average totN supported Chl-a during a second bloom in August, indicating that low water exchange and growth of slow-growing cyanobacteria within the water body may support Chl-a growth. Above-average totP concentrations during the onset of the first bloom acted as loss term on Chl-a, similar to totN, and further indicating a flushing effect of nutrient rich waters leading to suppression of even higher Chl-a growth. Above-average totP concentrations supported Chl-a in between both blooms, but acted as a loss term later in September.

None of the abiotic factors (water temperature, light attenuation, or salinity) were associated with active linear basis functions. The growth supporting quadratic salinity term was very weak, indicating that these coastal waters are mainly regulated by the amount of nutrients, or hydrodynamics. The most positive term during both blooms was $\text{totN}\cdot\text{Temp}$ also pointing to an interaction of nutrients and hydrodynamics, as discussed above the totN could come from outflow of nutrient rich waters from the catchment area with potentially higher temperature. Interestingly, $\text{totN}\cdot\text{Temp}$ also increased in importance for the blooming dynamics during the second bloom as the higher temperature during this period might play a more important role in the interaction term (cf. Table 1).

In summary, the nutrient status had the largest impact on the Advected Bloom, indirectly indicating an hydrodynamical impact. Future studies may need to include flow directions in these coastal waters, as they can change depending on wind fields, and precipitation.

There were 29 active basis functions identified for the **Delayed Spring Bloom**, the second highest number across all blooming patterns (Fig. 3, Fig. 4f).

Both dissolved nutrient fractions, DIN and DIP were associated with positive linear basis functions here. Above-average DIN supported Chl-a increase during the first bloom, and below-average concentrations suppressed Chl-a together with an always negative quadratic DIN term, indicating a potential high N-demand. This is in line with the fact that negative quadratic DIN term during the second bloom was the most negative (cf. Table 1). Similarly, below-average DIP was the most negative loss term for Chl-a during the first bloom (cf. Table 1), whereas above-average DIP concentrations supported the second bloom as one of the most positive terms. However, the effect of the growth supporting quadratic DIP term was very small. A N- and P-co-limitation has already been described for some of those coastal waters, explaining the impact of both DIN and DIP^54^. This described co-limitation is also in line with the interaction term of $\text{DIN}\cdot\text{DIP}$ as one of the most positive terms during both blooms (cf. Table 1).

Likewise, totN was an associated positive linear basis function and one of the most positive during the first bloom, supporting the Chl-a increase during the first bloom almost as strong as $\text{DIN}\cdot\text{DIP}$ (cf. Table 1). Below-average totN concentrations acted as a loss term on Chl-a during the second bloom. These results indicate a high N-demand, which is surprising considering the already high N:P ratio in these coastal waters^29^.

Interestingly, totP was associated with a negative linear basis function, with below-average totP concentrations supporting Chl-a during the first bloom. Similarly, above-average concentrations of totP suppressed Chl-a growth during the second bloom. These findings correlates with flushing in these innermost coastal waters, where most of the P is transported through rivers. An increased influx of river water during spring or summer can potentially flush out Chl-a and increases totP. There have been especially high precipitation events during summer months within the last two decades around the southern Baltic Sea coast^43,51^. This dynamic is consistent with the negative coefficient for the linear basis functions associated with salinity, leading to below-average salinity concentrations supporting Chl-a growth during the first bloom. Conversely, above-average salinity concentrations acted as a loss factor for Chl-a during the second bloom. The quadratic salinity term was always negative and growth-suppressing. These results indicate the overall dependency of inner coastal waters on nutrient supply through rivers, and dilution through intruding Baltic Sea water. However, alternatively phytoplankton growth is limited by totN leading to increased consumption of P and a population shift towards N-fixing cyanobacteria^54,55^.

The Kd light attenuation coefficient was associated with a positive linear basis function, but its impact throughout the year was negligible. This finding suggests that even in highly eutrophic coastal waters, light may not limit phytoplankton growth.

In contrast to all other blooms, the Delayed Spring Bloom had a constant growth term, indicating growth independent of all external drivers. This interaction coincided with their region of occurrence in the oligohaline estuarine lagoons with Chl-a concentrations above-average throughout the growth season. The constant growth was complemented by a quadratic loss term in Chl-a, indicating a regulation of Chl-a depending on the total amount of Chl-a available. However, this loss term was mainly weak and only active at the end of the first bloom.

In summary the Delayed Spring Bloom depended mostly on the amount of nutrients, as the most negative and positive terms are linked to nutrients (cf. Table 1). This dependency is somewhat surprising as these coastal waters show the highest nutrient concentrations across German coastal waters. Nonetheless, this dependency on nutrients also indicates that a strong decrease of nutrients may restore some of these systems back to a better state.

In sum, the diversity of interactions we found shows that dominant driving factors are different and differ in importance for the phytoplankton blooming pattern we examined.

## Conclusions and perspectives

The presented $\text {ANN}_\text {UDE}$ approach successfully learned different ecosystem dynamics in the form of phytoplankton blooming patterns, also described by the GAMM acting as spline-fitting function. Unlike the traditional GAMM, simultaneous learning of the dynamical relationship of input variables and their derivatives allowed the SInDy algorithm to unpack the learned dynamical relationships and describe them in an algebraic form. This approach enabled additional and interpretable insights into the dynamics of phytoplankton blooms in a data driven manner.

Remarkably, the relevant basis functions and their positive or negative coefficients represented known ecosystem processes, directly extracted from the monitoring data. This result highlights the ability of the approach to identify key drivers of the different blooming patterns. In the following we summarize the key findings and their implications for all blooming pattern:

For example, the growth supporting effect of temperature for the Outer Triplet Bloom can be explained by recent findings from longterm monitoring studies^40^. Furthermore, the strong impact of nutrient interactions, like $\text{DIN}\cdot\text{totP}$ highlight the importance of nutrient ratios in sustaining phytoplankton^27,45^. Thus, our approach can be used to model impacts of changing parameters on phytoplankton in such coastal waters. Nonetheless, the $\text {ANN}_\text {UDE}$ did not distinguish the second and third bloom event, even though they are described in literature^40^. Optimizing hyperparamter, ^e.g.^^56,57^ to balance the two terms in the LDA loss function, the SInDy sparsity threshold, and changes in the network architecture could improve the $\text {ANN}_\text {UDE}$ fit.

In contrast, the Inner Duplex Bloom was strongly impacted by DIN and totN, as well as the respective interactions of both nutrient fractions with salinity. Thus, from a management perspective, regulating N-inflows into these systems might be a viable option in regulating phytoplankton blooms.

A single summer bloom in coastal waters is rather unusual outside temperature-limited waters of northern latitudes^58^. The Summer Bloom was partly explained by an effect of temperature on Chl-a. However, this effect may have been indirect through the selection of cold-adapted diatoms during an early small phytoplankton bloom^40^, and an increased loss of Chl-a due to higher temperatures likely causing increased grazing activity during summer^49^, which is also in line with Chl-a dependent and constant loss. Coastal waters with summer blooms in this area can have extensive macrophyte stands^59^ which can support high zooplankton grazing rates^60^, potentially suppressing a spring and fall bloom. However, the apparent loss of grazing control during the summer requires further field studies. Furthermore, our results suggest, that again DIN is likely the growth-supporting factor for phytoplankton blooms in these coastal waters, especially during summer. However, future studies need to distinguish if the DIN signal stems from grazing on N-fixing cyanobacteria, or a release of DIN from sediments during potential anaerobic events in summer.

Interestingly, Chl-a growth for the Inner Triplet Bloom was supported if total nutrient concentrations were high and light limitation was low. These water bodies are located closer to nutrient-rich turbid rivers, and the open sea. Thus, within this mixing zone, an inflow of less turbid sea water could on the one hand decrease light limitation^61^ and through riverine inflow increase total available nutrients^50^. This dynamic is in contrast to the Outer Triplet Bloom, which also shows a three blooming period dynamic, but has different nutrient needs and seems to be more heavily regulated by salinity and temperature. The Inner Triplet Bloom is heavily impacted by amounts of DIN, as well as interactions of DIN with temperature, and DIN:DIP ratios. Our results suggests that again N-fractions need to be regulated in order to impact phytoplankton blooms.

Contrary, coastal waters with Advected blooms were mostly impacted by P-fractions, and through interaction terms including N-terms by N-fractions. Thus, our results suggest that P and N needs to be controlled in those coastal waters. However, there was a considerable differences between training and validation data sets. This difference could be explained by above long-term mean temperatures in 2019-2021 and below long-term precipitation in 2019 and 2020^43,62,63^. These conditions potentially decreased in- and outflow events in coastal waters governing the Advected Bloom^64,65^. The results also correlate with the fact that the Advected Bloom is dependent on nutrients entering and leaving the semi-enclosed coastal regions through flushing events. Missing precipitation and increasing temperature in a semi-enclosed coastal region would influence the occurrences of these flushing events and potentially change the blooming pattern.

Coastal waters with Delayed Spring Blooms were all part of oligohaline estuarine lagoons with above-average Chl-a concentrations throughout the growth season. Compared to all other blooming patterns, these coastal waters showed the widest distribution of nutrient coupled basis functions, where many N dependent combinations enhanced growth during blooms and $\text{DIN}\cdot\text{DIP}$ was the most positive term during both blooming periods. Additionally, the number of active basis functions coupled to salinity was highest for the Delayed Spring Bloom compared to other blooming patterns. This interaction could be interpreted in the context of significant impacts by riverine inflow^53,66^, precipitation^51^, or internal nutrient turnover and N-fixation even during light-limiting conditions^53,54^.

From a methodological point of view, there is still need for improvement of the $\text {ANN}_\text {UDE}$ approach.

The third bloom in the Inner Triplet Bloom was only represented by a weak shoulder at day 300 in the GAMM model and not modelled at all in the $\text {ANN}_\text {UDE}$. A reason could be potential changes in bloom timing and strength across years. Missing a bloom event can frequenently occur in coastal waters^67^. If the data set were dense enough the $\text {ANN}_\text {UDE}$ approach, applied to individual years with different bloom timing and strength, could potentially identify the driving factors for the occurrence or absence of a third bloom.

In general the $\text {ANN}_\text {UDE}$ approach was not able to fit individual yearly blooming dynamics in our case because of sparse data when considering single years. More densely sampled data per year should therefore enable learning a more accurate dynamical system that can account for variability across years. New methodological developments, such as those by Cheng et al.^9^, can also enable machine learning models to learn dynamics from sparser data with appropriate constraints.

Additionally, an ensemble fit with multiple $\text {ANN}_\text {UDE}$ runs was chosen in this work to deal with training variability and give robust estimates of method performance. In future work, this approach can also inform better SInDy estimations of basis functions and include, for example, distributions for each parameter. Ensemble $\text {ANN}_\text {UDE}$ should thus be combined with approaches like ensemble-SInDy^68^.

An other aspect is the choice of the basis function library, because this can impact the algebraic description of the relationship between model variables and external drivers. In our case, we opted for polynomials up to second order as Taylor expansion approximation of more complex interactions. It is also possible to use higher order polynomials including the risk of overfitting or directly incooperate for example Holling-type interactions into the basis function library and with this knowledge restrict the algebraic description. Here we refrained from doing so in order to influence the dynamics as little as possible. Future work can either inform the Universal Differential Equation guess (Box ”Universal Differential Equation” in blue path Fig. 1b) by incooperating previous knowledge on interactions into the equation or expand the basis function library by functions commonly used in phytoplankton bloom modelling. Other approaches like symbolic regression, ^e.g.^^69^ could also guide the choice of the basis function library.

All these aspects will not only improve the quality of the model fit, but also further increase the interpretability of the results.

Revisiting our overarching methodological question from the introduction, we can state, that we can validated known ecosystem observations in a model directly derived from monitoring data of a diverse set of coastal waters and also identify effects not directly measured in the data. We did not have to make any assumptions beyond some interactions of nutrients and chlorophyll-a and the potential impact of external drivers. The extracted couplings agreed surprisingly well with known ecological explanations on the interactions in the system as discussed above, but also uncovered different interactions for different phytoplankton blooming patterns.

For management actions along the Baltic Sea coast, these data-driven, bottom-up insights can help improve region- and case-specific actions that cannot be derived from general, knowledge-driven, top-down assumptions of growth and loss terms alone. Identifying strongest positive and negative contributing terms for the blooming period as we did in Table 1 may serve as the starting point for management action discussions. We showed that blooms would react differently to nutrient reduction due to different direct and indirect dependencies and possible additional hydrodynamical impact. Our analyses also provides insights into temperature driven changes, as the Baltic Sea warms faster than the global mean^26,70^. We can clearly identify coastal waters where Chl-a was positively (growth supporting) impacted (Outer Triplet Bloom) or negatively (growth suppressing) impacted (Inner Duplex Bloom and Summer Bloom) by increased temperature. In a future step the found temperature impact can be related to changing phytoplankton community compositions and risk assessments.

The presented approach is not limited to the presented region or data set but can be applied and adapted where ever sufficiently dense data is sampled for at least one blooming period. Also the selection of differential equation variables like Chl-a, DIN, DIP and external drivers can be adapted to the investigated bloom. It is also possible to reconstruct species specific dynamical descriptions if species measurements are available or to include predators like zooplankton if data is available.

Therefore, combining knowledge-based and data-driven modelling can significantly improve our understanding of phytoplankton blooming dynamics and our ability to construct models that can predict their development even on smaller regional scales.

## Supplementary Information

Supplementary Information.

## Data Availability

The data and the code used to calculate the results in this work are available at https://github.com/pnieters/PredictingPhytoplanktonPatterns. All data presented as results are directly reproduceable from the code, all data this work is based on is available in the preprocessed format we used. The raw data was supplied by the State Agency for Environment, Nature Conservation and Geology Mecklenburg-Vorpommern (LUNG-MV).
