# Supplementary material for: Machine learning to identify environmental drivers of phytoplankton blooms in the Southern Baltic Sea
Source: Sci Rep. 2025 Jan 24;15:3077. doi: 10.1038/s41598-025-85605-y (PMC11760947; doi:10.1038/s41598-025-85605-y)
Supplement: Supplementary file 1 — Supplementary Information. [file 41598_2025_85605_MOESM1_ESM.pdf]

Supplementary Material to: Machine Learning to Identify Environmental Drivers of Phytoplankton Blooms in the Southern Baltic Sea

Maximilian Berthold[1,+], Pascal Nieters[2, +], and Rahel Vortmeyer-Kley[3,*,+]

[1]Department of Biology, Faculty of Science, Mount Allison University, Canada
[2]Institute of Cognitive Science, Osnabrück University, Germany
[3]Institute for Chemistry and Biology of the Marine Environment, Carl von Ossietzky University Oldenburg, Germany
[*]rahel.vortmeyer-kley@uni-oldenburg.de
[+]these authors contributed equally to this work

ABSTRACT

This supplementary material contains additional information and figures to the paper: "Machine Learning to Identify Environmental Drivers of Phytoplankton Blooms in the Southern Baltic Sea"

Phytoplankton blooms exhibit varying patterns in timing and number of peaks within ecosystems. These differences in blooming patterns are partly explained by phytoplankton:nutrient interactions and external factors such as temperature, salinity and light availability. Understanding these interactions and drivers is essential for effective bloom management and modelling as driving factors potentially differ or are shared across ecosystems on regional scales. Here, we used a 22-year data set (19 years training and 3 years validation data) containing chlorophyll, nutrients (dissolved and total), and external drivers (temperature, salinity, light) of the southern Baltic Sea coast, a European brackish shelf sea, which constituted six different phytoplankton blooming patterns. We employed generalized additive mixed models to characterize similar blooming patterns and trained an artificial neural network within the Universal Differential Equation framework to learn a differential equation representation of these pattern. Applying Sparse Identification of Nonlinear Dynamics uncovered algebraic relationships in phytoplankton:nutrient:external driver interactions. Nutrients availability was driving factor for blooms in enclosed coastal waters; nutrients and temperature in more open regions. We found evidence of hydrodynamical export of phytoplankton, natural mortality or external grazing not explicitly measured in the data. This data-driven workflow allows new insight into driver-differences in region specific blooming dynamics.
keywords: Scientific machine learning & Sparse Identification of nonlinear Dynamics & General Additive Mixed Model & Baltic Sea & phytoplankton blooms

S1 External drivers: temperature, salinity and light attenuation

The functional form of the external drivers is a polynomial fit of the dynamics the log-transformed and z-scored data of temperature (Fig.S1), salinity (Fig.S2) and light attenuation Kd (Fig.S3) for each phytoplankton blooming pattern for the years 2000-2018 together. The polynomial fits represent a mean-behavior on a day-of-the-year scale for all training years together. The polynomial fit for water temperature is of the form: $y(t) = \sum_{n=0}^{5} p_n \cdot x^n$ and for salinity and Kd of the form $y(t) = \sum_{n=0}^{4} p_n \cdot x^n$.
How to build the polynomials and their parameters are documented in the code FitDriverFunctionTempSalKd.m at `https://github.com/pnieters/PredictingPhytoplanktonPatterns`.

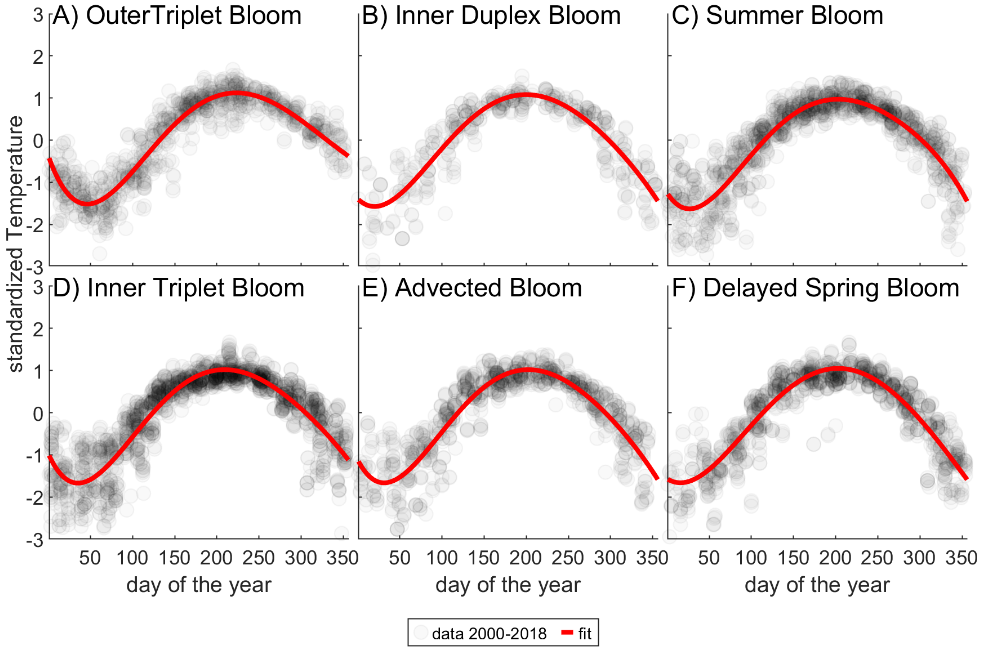

Figure S1. Standardized temperature dynamics of the different blooming patterns: A) Outer Triplet Bloom, B) Inner Duplex Bloom, C) Summer Bloom, D) Inner Triplet Bloom, E) Advected Bloom, F) Delayed Spring Bloom. Light gray dots: log-transformed and z-scored data of temperature measured at the 30 stations between 2000-2018. red: polynomial fit of order five.

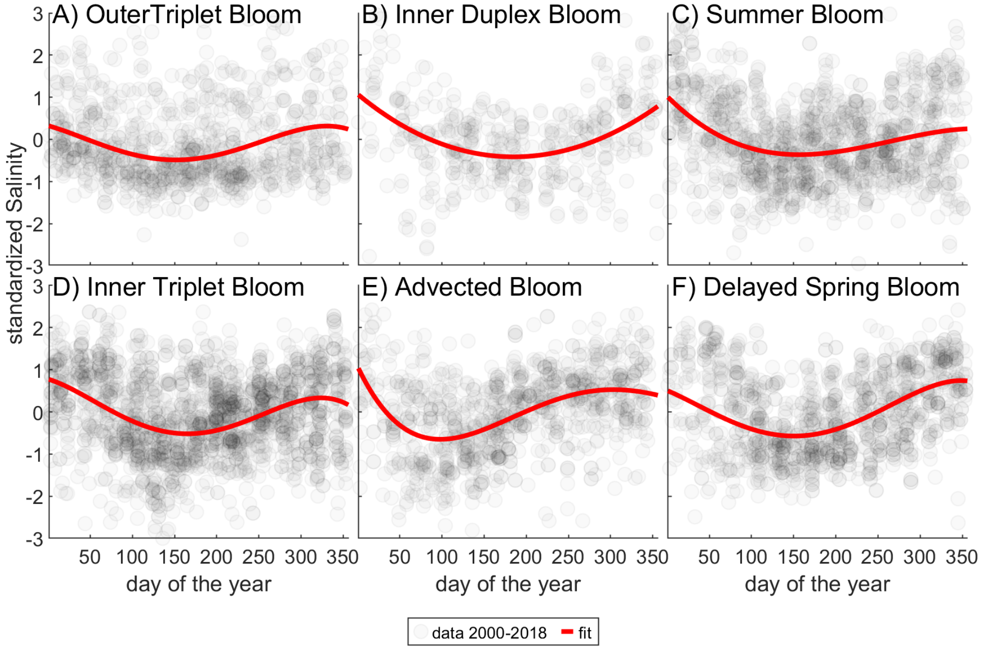

Figure S2. Standardized salinity dynamics of the different blooming patterns: A) Outer Triplet Bloom, B) Inner Duplex Bloom, C) Summer Bloom, D) Inner Triplet Bloom, E) Advected Bloom, F) Delayed Spring Bloom. Light gray dots: log-transformed and z-scored data of temperature measured at the 30 stations between 2000-2018. red: polynomial fit of order four.

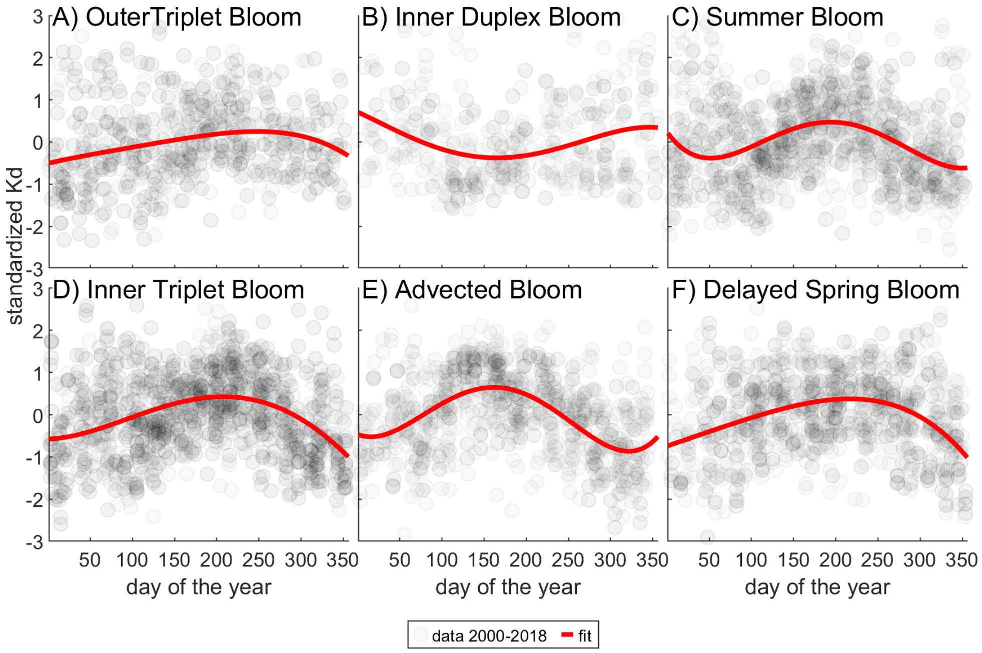

Figure S3. Standardized light attenuation Kd dynamics of the different blooming patterns: A) Outer Triplet Bloom, B) Inner Duplex Bloom, C) Summer Bloom, D) Inner Triplet Bloom, E) Advected Bloom, F) Delayed Spring Bloom. Light gray dots: log-transformed and z-scored data of temperature measured at the 30 stations between 2000-2018. red: polynomial fit of order four.

S2 Comparison model and data

Table S1 show the MSE values for the comparison of both models and chlorophyll data as well as model-model comparison for the training data set 2000-2018. Table S2 show the MSE values for the comparison of both models and chlorophyll data for the validation data set 2019-2021.

Table S1. Model-data and model-model comparison according to Eq. 1 and Eq. 2 for chlorophyll training data set 2000-2018

**blooming pattern**	$\mathbf{MSE_{ANN-data}}$	$\mathbf{MSE_{GAMM-data}}$	$\mathbf{MSE_{GAMM-ANN}}$
Outer Triplet Bloom	0.67	0.62	0.10
Inner Duplex Bloom	0.83	0.81	0.04
Summer Bloom	0.80	0.74	0.08
Inner Triplet Bloom	0.67	0.66	0.05
Advected Bloom	0.92	0.84	0.11
Delayed Spring Bloom	0.77	0.70	0.08

Table S2. Model-data comparison according to Eq. 1 and Eq. 2 for chlorophyll validation data set 2019-2021

**blooming pattern**	$\mathbf{MSE_{ANN-data}}$	$\mathbf{MSE_{GAMM-data}}$
Outer Triplet Bloom	0.66	0.51
Inner Duplex Bloom	1.26	1.37
Summer Bloom	1.21	1.16
Inner Triplet Bloom	1.08	1.14
Advected Bloom	2.46	1.94
Delayed Spring Bloom	1.33	1.19

S3 Sign of interaction terms

In general, terms (coefficient·variables) with a positive overall sign can be considered as growth terms leading to an increase of Chl-a, while terms with a negative overall sign can be interpreted as loss terms (potential limitation, or mortality) leading to a decrease of Chl-a.
A positive overall sign results from a negative coefficient combined with a negative log-transformed z-score of the variables, or from a positive coefficient combined with a positive log-transformed z-score of the variables. A negative overall sign results from a negative coefficient combined with a positive log-transformed z-score of the variables, or from a positive coefficient combined with a negative log-transformed z-score of the variables. For a product of several variables the overall sign of the product combined with the coefficient gives the overall sign of the term. Table S3 displays all possible combinations of signs of the variables and coefficients and their effects as growth or loss term.

Table S3. Overall sign of term coefficient·variables and its effect

sign coefficient	sign variable 1	sign variable 2	overall sign (effect)
-	-	/	+ (growth)
-	+	/	- (loss)
-	-	-	- (loss)
-	+	+	- (loss)
-	+	-	+ (growth)
+	-	/	- (loss)
+	+	/	+ (growth)
+	-	-	+ (growth)
+	+	+	+ (growth)
+	+	-	- (loss)

The sign of variables is understood as complete sign. Variable 1 can also be the same as variable 2, resulting in $(\text{variable } 1)^2$.

S4 Results of 20 runs of ANN

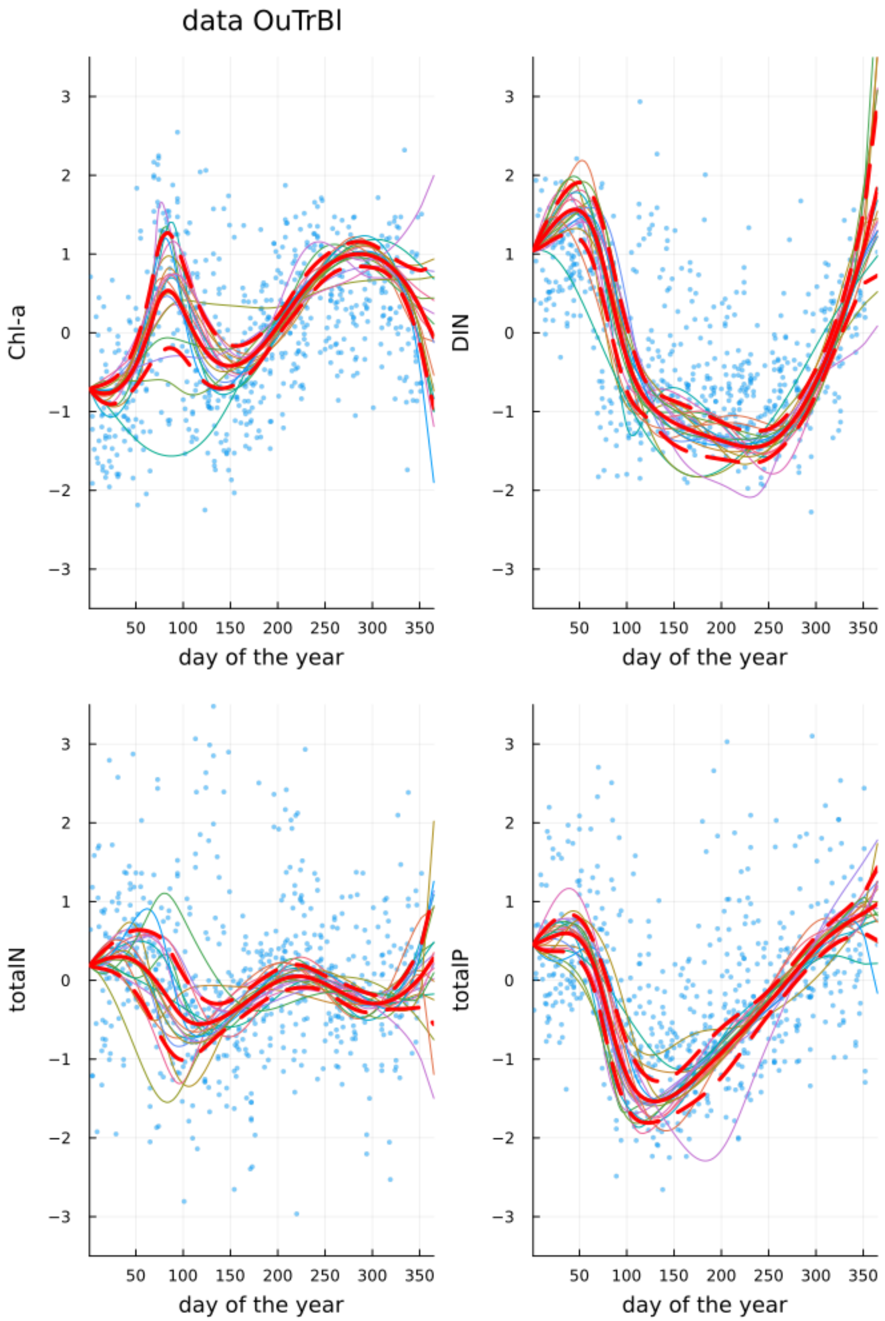

Figure S4. Results for all 20 runs of the ANN for **Outer Triplet Bloom** data 2000-2018 (blue dots). Red bold line: mean model, red dashed lines: standard deviation. The y axis are given as standardized values. Used external drivers are temperature, salinity and light attenuation.

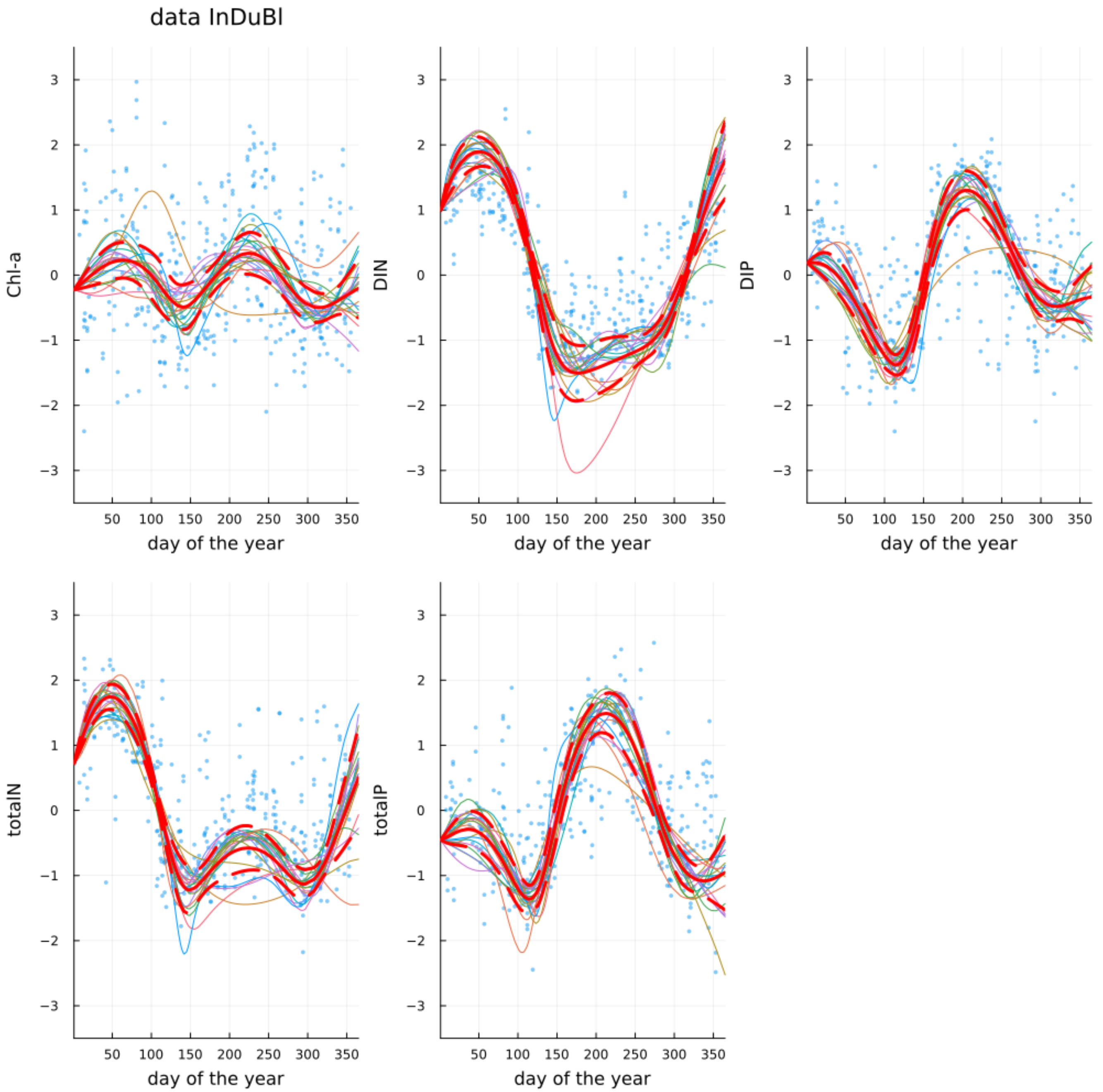

Figure S5. Results for all 20 runs of the ANN for **Inner Duplex Bloom** data 2000-2018 (blue dots). Red bold line: mean model, red dashed lines: standard deviation. The axis y are given as standardized values. Used external drivers are temperature, salinity and light attenuation.

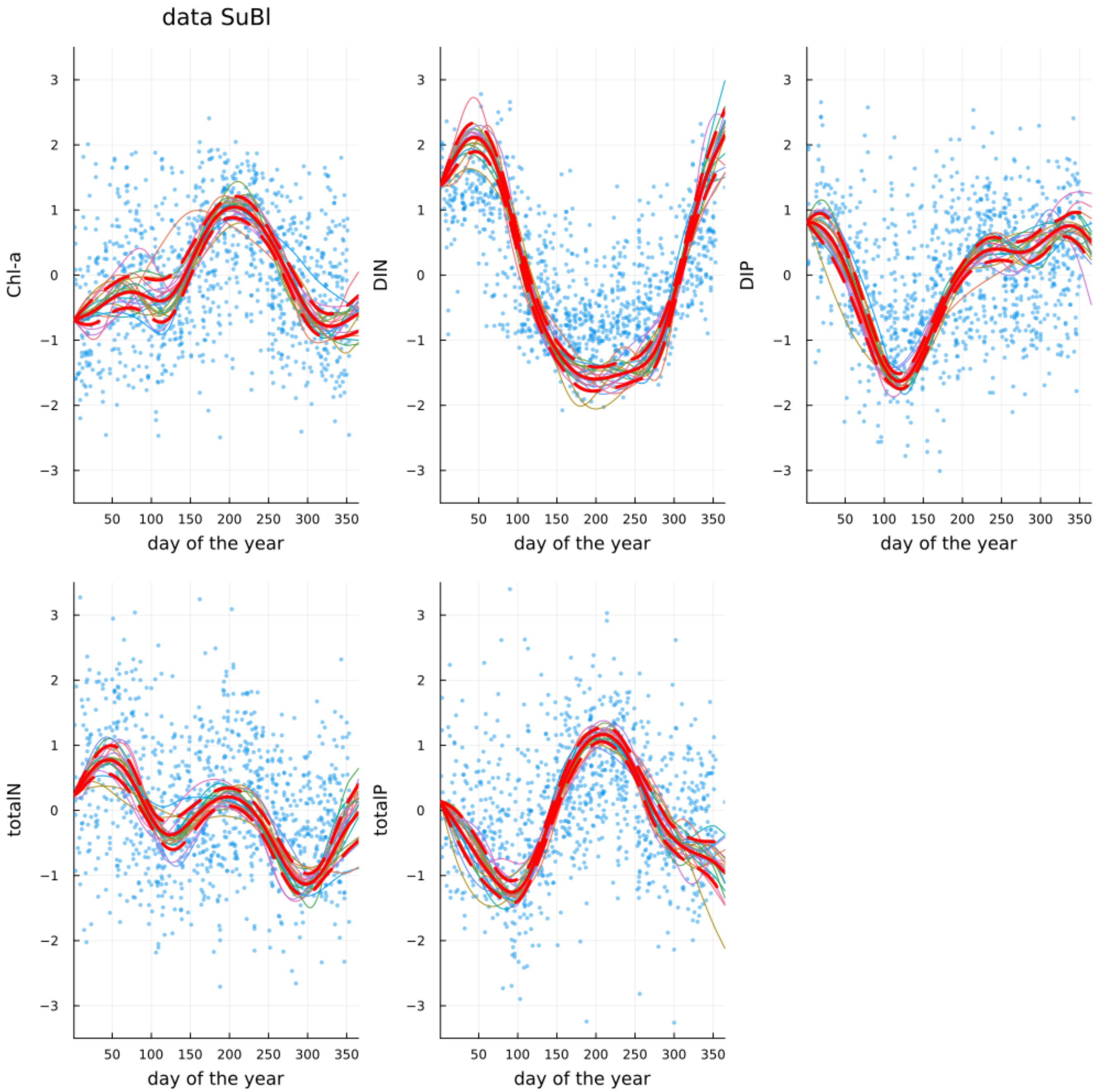

Figure S6. Results for all 20 runs of the ANN for **Summer Bloom data** 2000-2018 (blue dots). Red bold line: mean model, red dashed lines: standard deviation. The y axis are given as standardized values. Used external drivers are temperature, salinity and light attenuation.

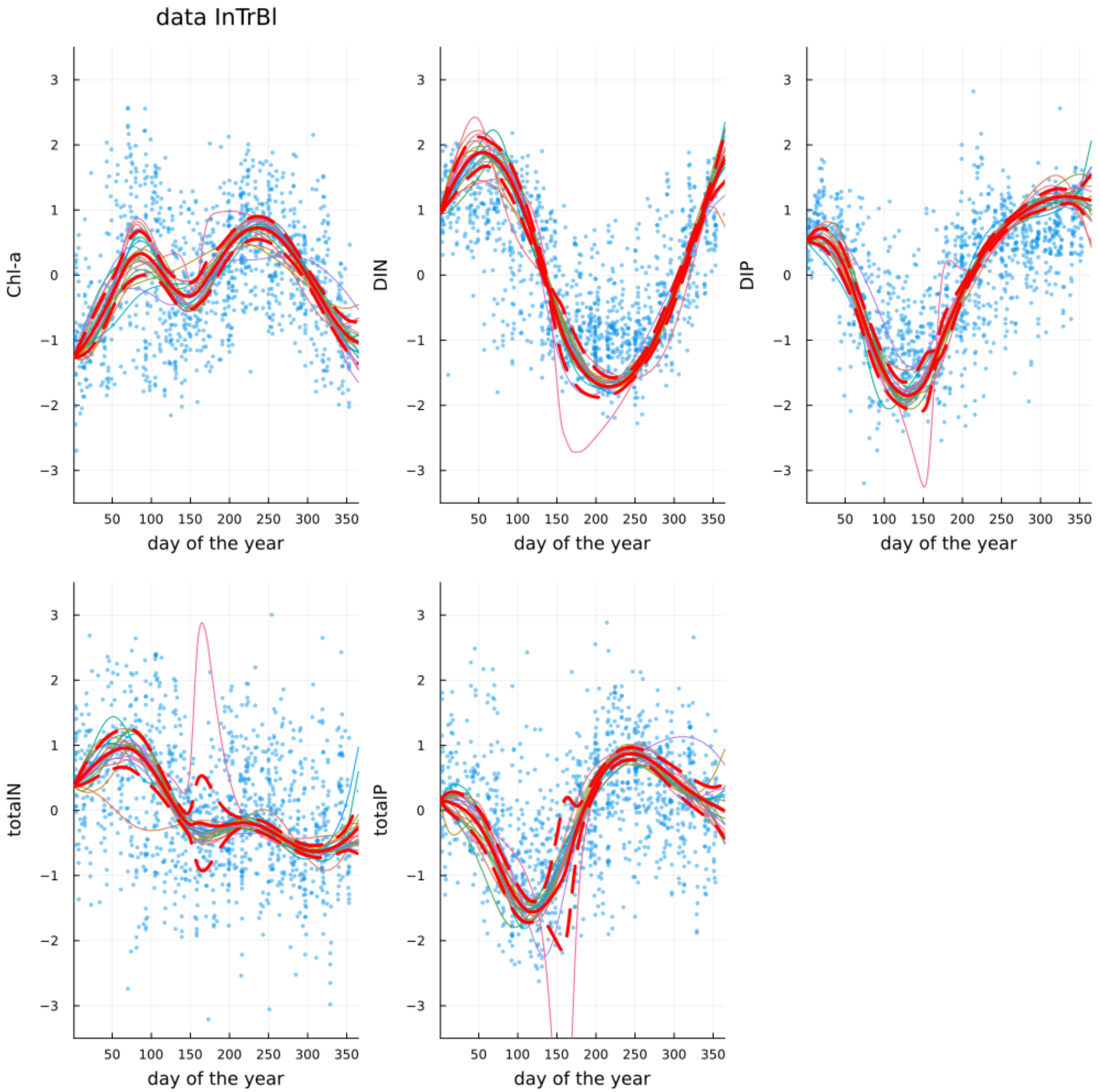

Figure S7. Results for all 20 runs of the ANN for **Inner Triplet Bloom data** 2000-2018 (blue dots). Red bold line: mean model, red dashed lines: standard deviation. The y axis are given as standardized values. Used external drivers are temperature, salinity and light attenuation.

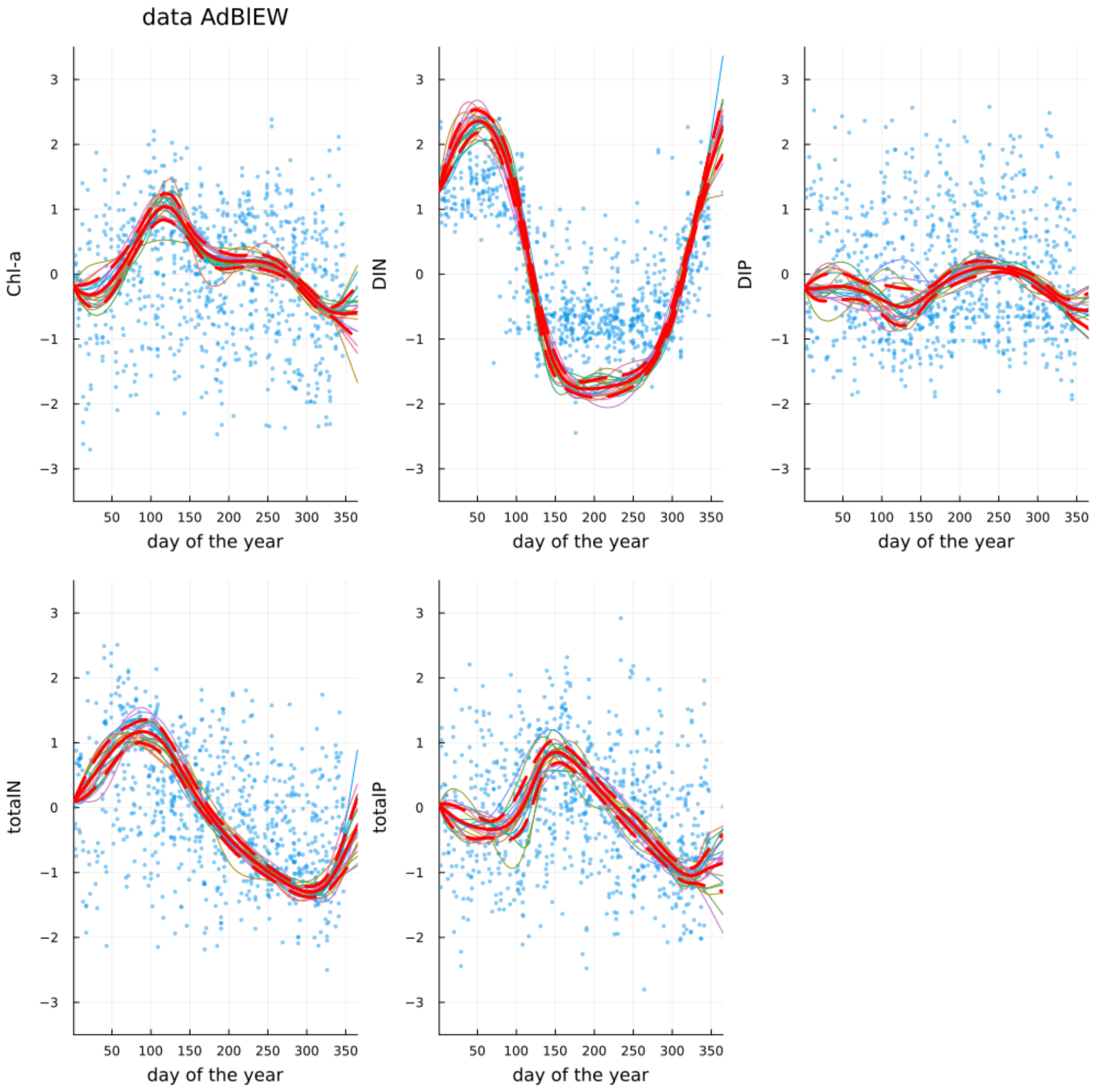

Figure S8. Results for all 20 runs of the ANN for **Advected Bloom data** 2000-2018 (blue dots). Red bold line: mean model, red dashed lines: standard deviation. The y axis are given as standardized values. Used external drivers are temperature, salinity and light attenuation.

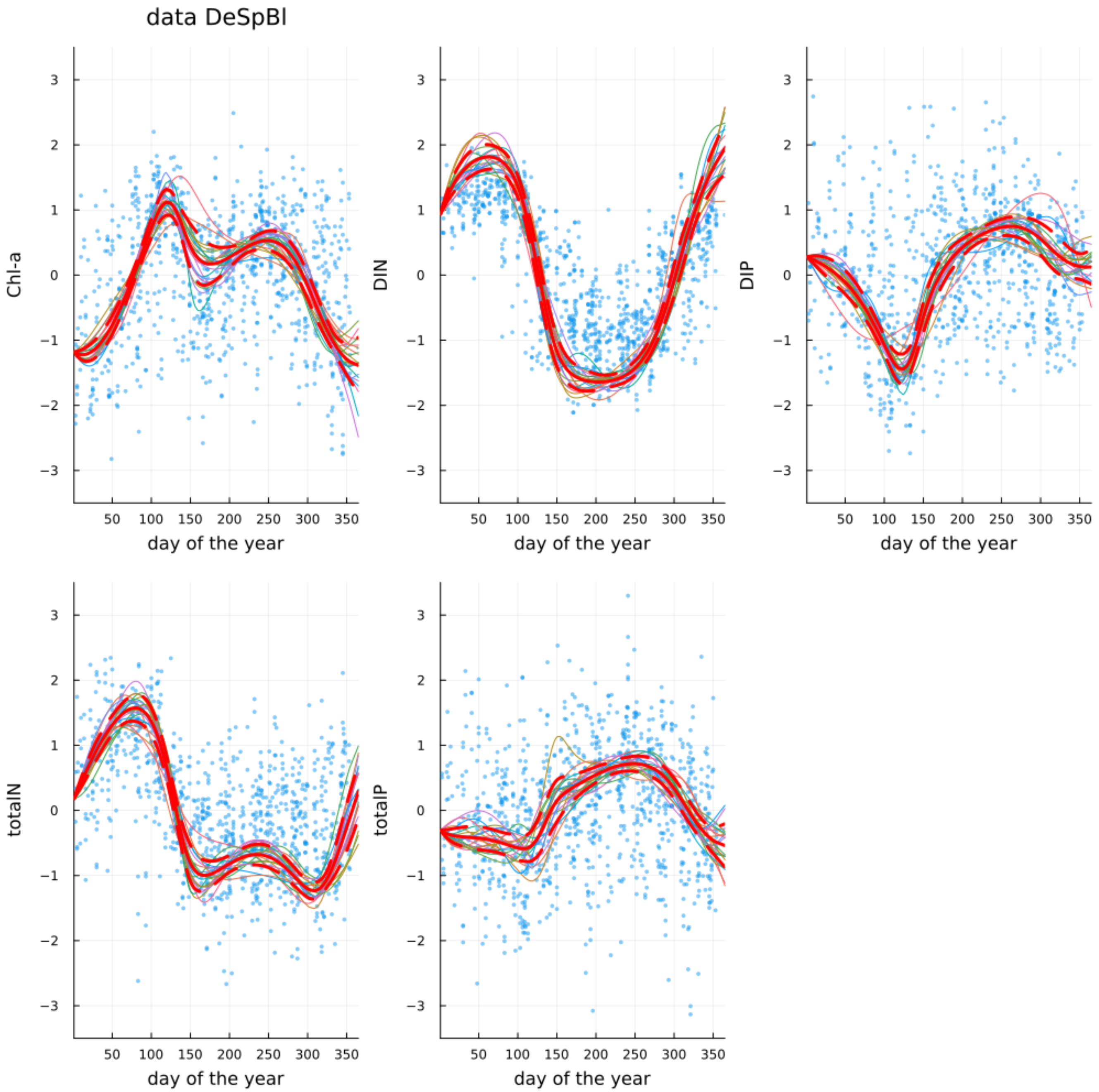

Figure S9. Results for all 20 runs of the ANN for **Delayed Spring Bloom** data 2000-2018 (blue dots). Red bold line: mean model, red dashed lines: standard deviation. The y axis are given as standardized values. Used external drivers are temperature, salinity and light attenuation.

S5 Mean model

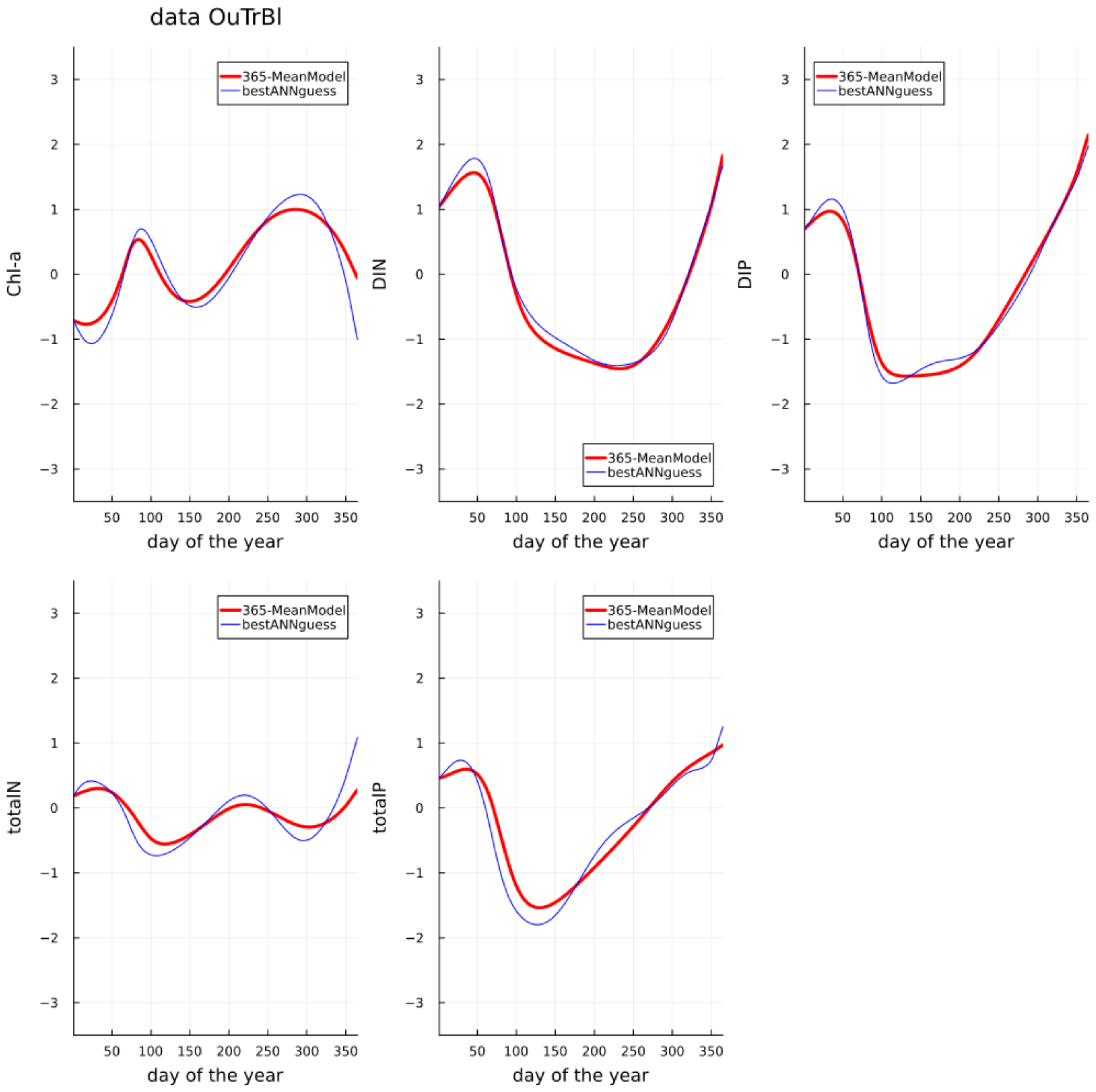

Figure S10. Mean Model for Outer Triplet Bloom (red bold line) and selected run for SInDy reconstruction (blue thin line). The y axis are given as standardized values.

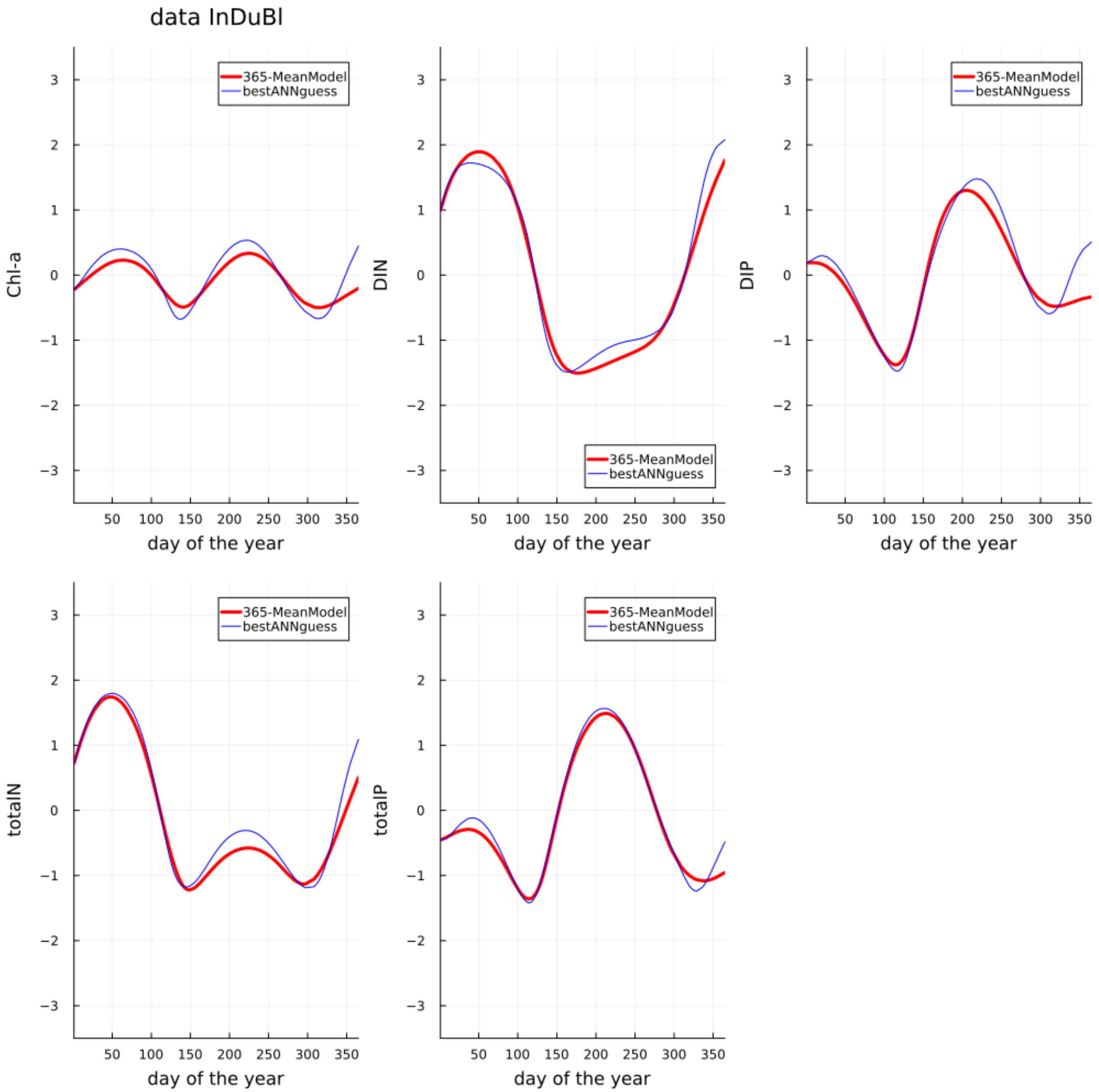

Figure S11. Mean Model for Inner Duplex Bloom (red bold line) and selected run for SInDy reconstruction (blue thin line). The y axis are given as standardized values. Used external drivers are temperature, salinity and light attenuation.

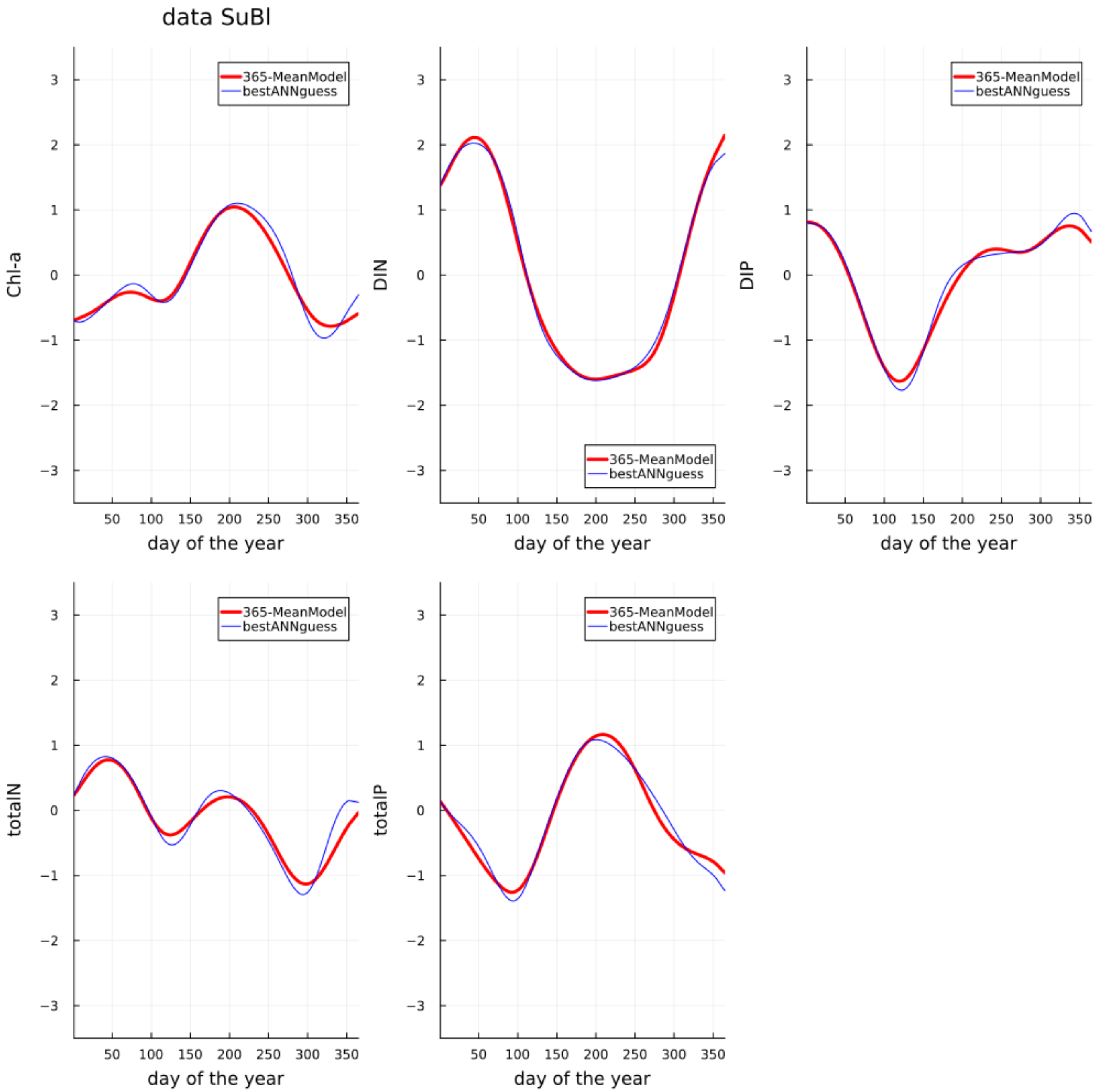

Figure S12. **Mean Model for Summer Bloom** (red bold line) and selected run for SInDy reconstruction (blue thin line). The y axis are given as standardized values. Used external drivers are temperature, salinity and light attenuation.

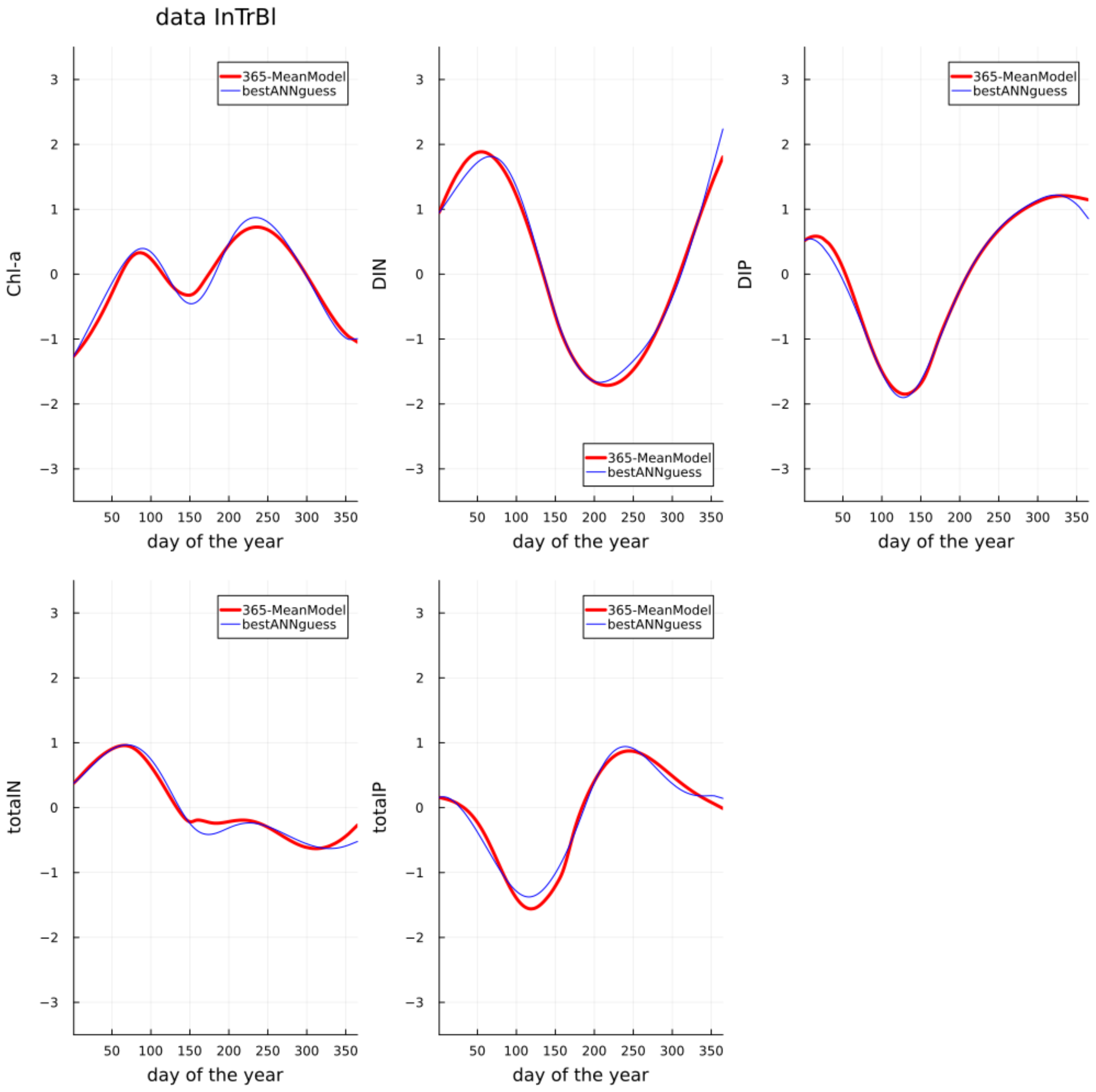

Figure S13. Mean Model for Inner Triplet Bloom (red bold line) and selected run for SInDy reconstruction (blue thin line). The y axis are given as standardized values.

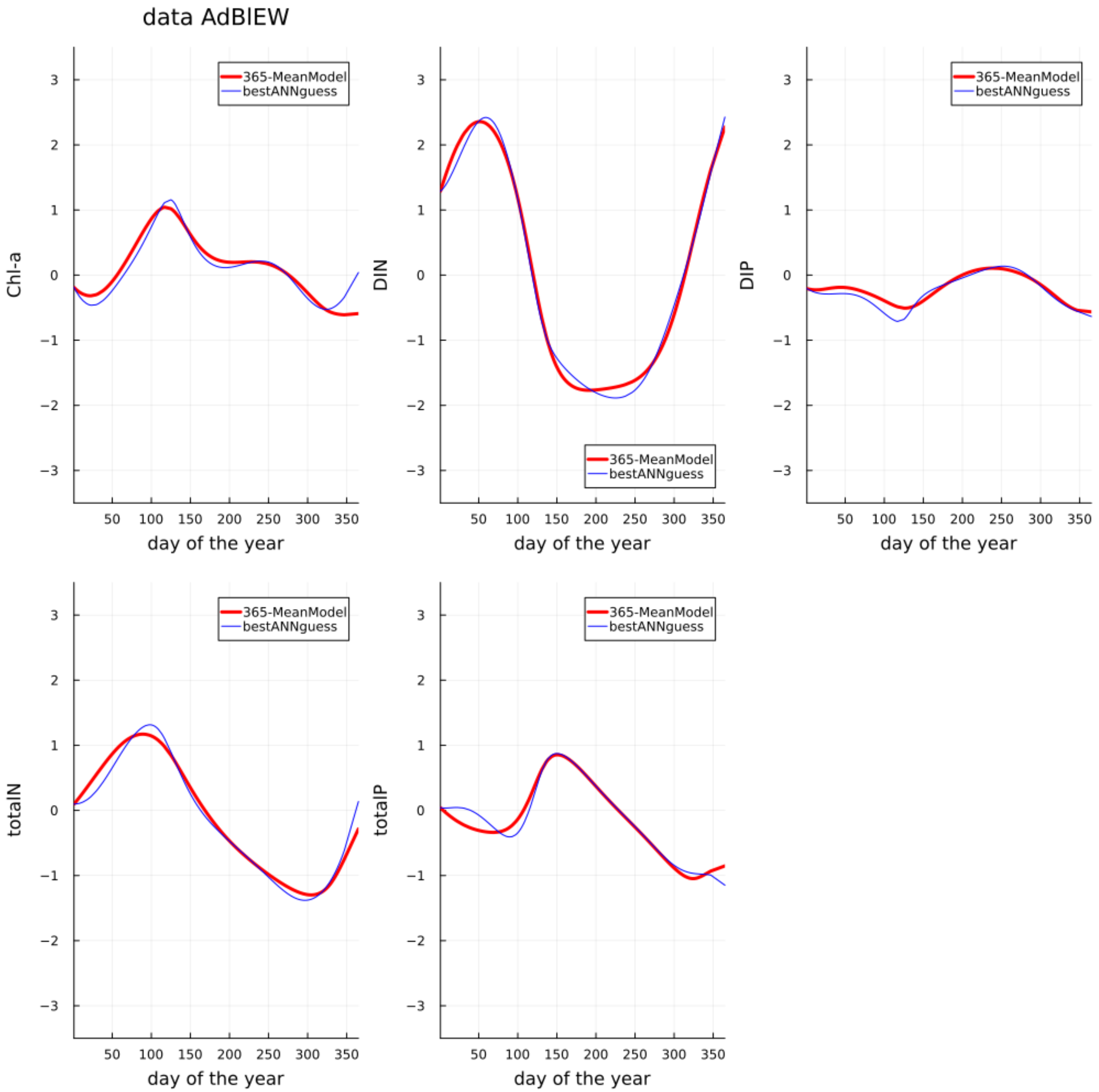

Figure S14. **Mean Model for Advected Bloom** (red bold line) and selected run for SInDy reconstruction (blue thin line). The y axis are given as standardized values. Used external drivers are temperature, salinity and light attenuation.

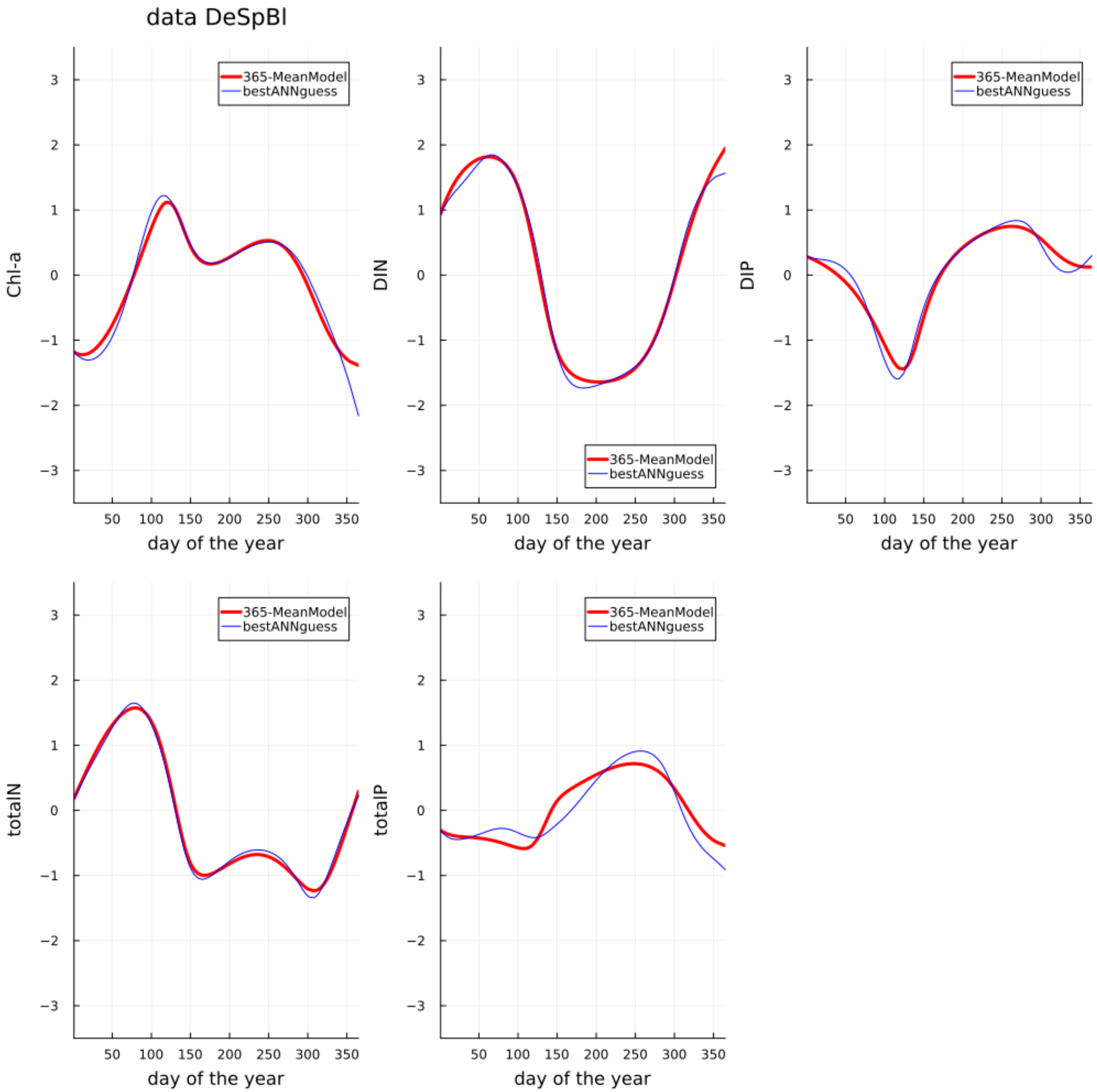

Figure S15. **Mean Model for Delayed Spring Bloom** (red bold line) and selected run for SInDy reconstruction (blue thin line). The y axis are given as standardized values. Used external drivers are temperature, salinity and light attenuation.

S6 Recovered differential equations for the different phytoplankton blooming patterns

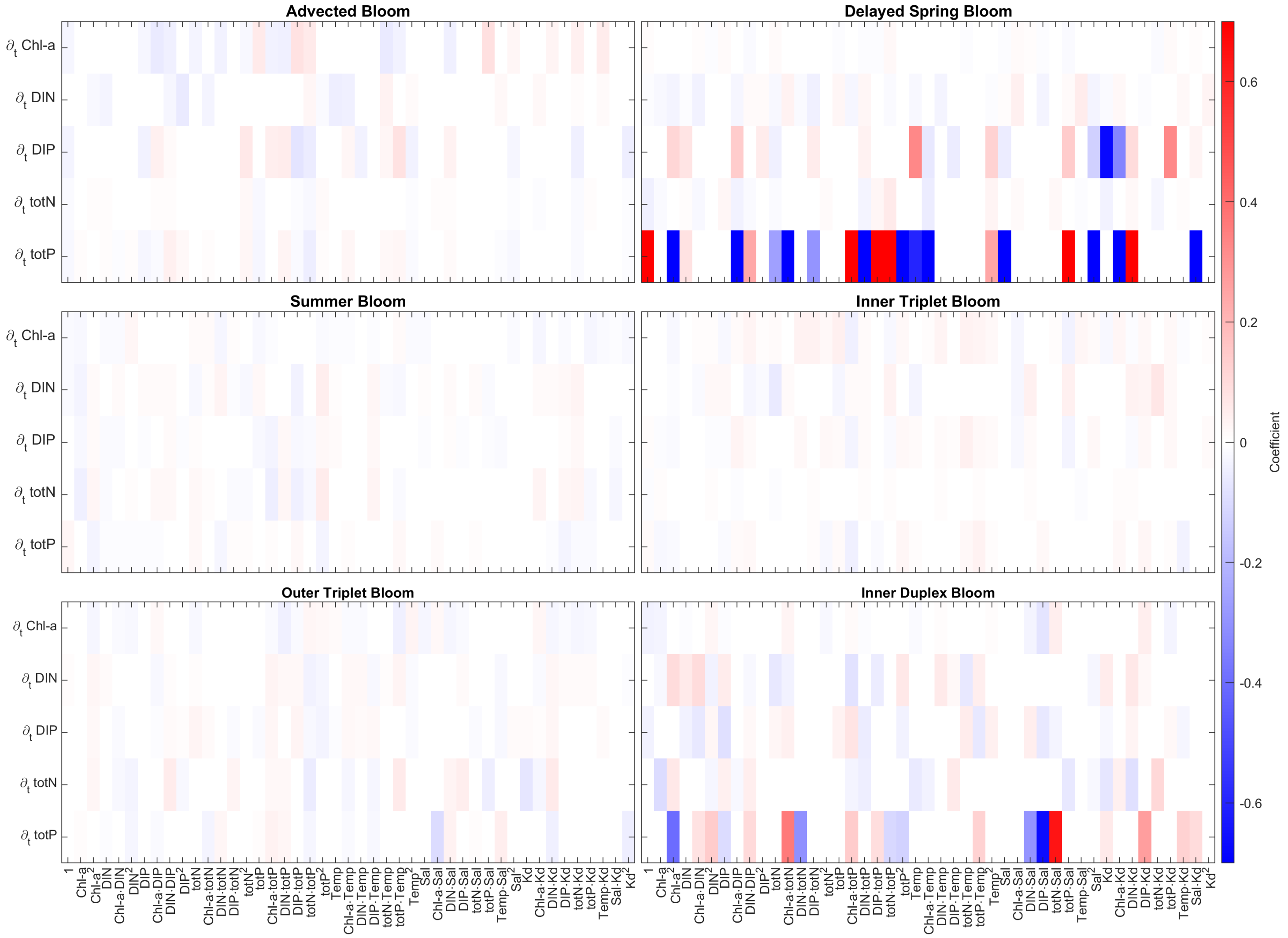

Figure S16. Graphical representation of by SInDy recovered differential equations for the different phytoplankton blooming patterns. The x axis represent the basis functions, the y axis set of differential equations and the colorcoded entries the value of the parameters that are multiplied with the respective basis function. Used external drivers are temperature, salinity and light attenuation. For better visualization the coloraxis of the coefficients is cut at ± 1.72. The largest coefficient is 8.69 and the smallest -11.01 (both Delayed Spring Bloom). The full set of equations including the values of the coefficients presented in Fig. S16 can be found in SindyOUTallEQ.txt at `https://github.com/pnieters/PredictingPhytoplanktonPatterns`.
